# The *Neurospora* Transcription Factor ADV-1 Transduces Light Signals and Temporal Information to Control Rhythmic Expression of Genes Involved in Cell Fusion

**DOI:** 10.1534/g3.116.034298

**Published:** 2016-11-15

**Authors:** Rigzin Dekhang, Cheng Wu, Kristina M. Smith, Teresa M. Lamb, Matthew Peterson, Erin L. Bredeweg, Oneida Ibarra, Jillian M. Emerson, Nirmala Karunarathna, Anna Lyubetskaya, Elham Azizi, Jennifer M. Hurley, Jay C. Dunlap, James E. Galagan, Michael Freitag, Matthew S. Sachs, Deborah Bell-Pedersen

**Affiliations:** * Department of Biology, Texas A&M University, College Station, Texas 77843; † Department of Biochemistry and Biophysics, Oregon State University, Corvallis, Oregon 97331; ‡ Bioinformatics Program, Boston University, Massachusetts 02215; § Department of Genetics, Geisel School of Medicine at Dartmouth, Hanover, New Hampshire 03755; ** Department of Biological Sciences, Rensselaer Polytechnic Institute, Troy, New York 12180; †† National Emerging Infectious Diseases Laboratories, Boston University, Massachusetts 02118; ‡‡ Department of Microbiology, Boston University, Massachusetts 02215; §§ Department of Biomedical Engineering, Boston University, Massachusetts 02215

**Keywords:** phototransduction, circadian clock, development, regulatory network

## Abstract

Light and the circadian clock have a profound effect on the biology of organisms through the regulation of large sets of genes. Toward understanding how light and the circadian clock regulate gene expression, we used genome-wide approaches to identify the direct and indirect targets of the light-responsive and clock-controlled transcription factor ADV-1 in *Neurospora crassa*. A large proportion of ADV-1 targets were found to be light- and/or clock-controlled, and enriched for genes involved in development, metabolism, cell growth, and cell fusion. We show that ADV-1 is necessary for transducing light and/or temporal information to its immediate downstream targets, including controlling rhythms in genes critical to somatic cell fusion. However, while ADV-1 targets are altered in predictable ways in Δ*adv-1* cells in response to light, this is not always the case for rhythmic target gene expression. These data suggest that a complex regulatory network downstream of ADV-1 functions to generate distinct temporal dynamics of target gene expression relative to the central clock mechanism.

Circadian clocks, comprised of molecular oscillators, provide a mechanism for organisms to coordinate their physiology and behavior with daily environmental cycles, and to maintain internal temporal order (Bell-Pedersen *et al.* 2005). External cues, particularly light signals, entrain clocks to 24-hr environmental cycles. As such, light and circadian clock signaling pathways are closely intertwined. Temporal information is relayed from circadian oscillators through output pathways to regulate daily rhythms in the expression of clock-controlled genes (*ccgs*) and overt rhythmicity. While substantial progress has been made in understanding the central clock machinery (Crane and Young 2014; Bell-Pedersen *et al.* 2005; Siepka *et al.* 2007; Gustafson and Partch 2015; Hardin 2011; Dunlap *et al.* 2007) and cataloging rhythmic and light-responsive genes (Duffield 2003; Hardin 2000; Pfeiffer *et al.* 2014; Vitalini *et al.* 2006; Chen *et al.* 2009; Wu *et al.* 2014; Hurley *et al.* 2014; Zhang *et al.* 2014; Rodriguez *et al.* 2013; Sancar *et al.* 2015b), an important remaining challenge is to connect the circadian oscillator to the output pathways to determine how the clock regulates rhythms in biological processes, including development, cell growth, and metabolism.

*Neurospora crassa* is one of the best-studied model systems for light responses and circadian rhythms (Vitalini *et al.* 2010). The core *N. crassa* FRQ/WCC circadian oscillator forms a characteristic negative feedback loop. In the FRQ/WCC oscillator, two PAS-domain-containing GATA-type zinc finger transcription factors (TFs), White Collar-1 (WC-1) and White Collar-2 (WC-2), dimerize to form the White Collar Complex (WCC) (Ballario *et al.* 1996; Denault *et al.* 2001; Cheng *et al.* 2002). WCC functions as a positive element in the oscillator and activates transcription of the *frequency* (*frq*) gene (Froehlich *et al.* 2002; Vitalini *et al.* 2010; Lee *et al.* 2003). The negative component FRQ accumulates, enters the nucleus, interacts with FRH (FRQ interacting RNA helicase) (Cheng *et al.* 2005; Shi *et al.* 2010) and CK1 (Baker *et al.* 2009), and inhibits the activity of WCC (He *et al.* 2005; Schafmeier *et al.* 2005, 2008). Progressive phosphorylation of FRQ relieves WCC inhibition, reinitiates the cycle, and leads to proteasome-dependent degradation of FRQ (Liu *et al.* 2000; He *et al.* 2003; Larrondo *et al.* 2015).

WC-1 is also a blue light photoreceptor (Cheng *et al.* 2003; He *et al.* 2002; Froehlich *et al.* 2002), and with its partner WC-2, functions to regulate light-responsive genes, as well as downstream *ccgs* (Chen *et al.* 2009; Smith *et al.* 2010). Upon light exposure, the WCC binds to light-responsive elements in the promoters of target light-responsive genes (Froehlich *et al.* 2002; Smith *et al.* 2010; Olmedo *et al.* 2010; He *et al.* 2005). Recent RNA-seq studies revealed that at least 31% of expressed genes in *N. crassa* are regulated by light (Wu *et al.* 2014). In addition, ChIP-seq in cells given a short light pulse to activate the WCC revealed that WCC binding increased at ∼400 sites in the genome, including at the promoters of ∼200 genes (Smith *et al.* 2010). Genes encoding TFs (24 TF genes) were enriched among these direct WCC targets. ChIP-seq data supported expression studies suggesting a flat hierarchical network in which light-activated WCC directly controls the expression of early light-induced genes, including some TF genes (Chen *et al.* 2009). These early light-induced TFs would in turn control the expression of late light-responsive genes. Consistent with this model, the *sub-1* gene, encoding a GATA-family TF, is an early light-induced gene whose promoter is bound by the WCC, and deletion of *sub-1* alters light responses for many late light-inducible genes (Chen *et al.* 2009; Smith *et al.* 2010; Sancar *et al.* 2015a). Furthermore, regulatory hierarchy can also explain evening-phase-specific gene expression. The WCC activates the expression of *csp-1*, which functions mainly as a repressor, and as a result turns off its targets in the morning phase (Sancar *et al.* 2011; Lambreghts *et al.* 2009). However, how the other 22 identified TFs are linked to light signaling and/or circadian output pathways is not known. This information is necessary for generating models that will comprehensively describe the light and circadian TF network that will be useful for developing predictions concerning time-of-day-dependent biological reactions. Such studies could potentially lead to the identification of drug targets to help humans with impaired or chronically desynchronized clocks.

The *arrested development-1* (*adv-1*; NCU07392) gene promoter is bound by the WCC (Smith *et al.* 2010). ADV-1 is a Zn(II)_2_Cys_6_ zinc cluster TF necessary for vegetative and sexual development in *N. crassa* (Colot *et al.* 2006), and is homologous to PRO1 in *Sordaria macrospora*, which is required for fruiting body development (Masloff *et al.* 1999). Deleting *adv-1* impairs the formation of protoperithecia, the precursor to female reproductive structures (perithecia), rendering the strain female sterile (Colot *et al.* 2006). Consistent with this phenotype, Δ*adv-1* cells lack conidial anastomosis tubes (CATs) that facilitate somatic cell fusion during conidial germination and during hyphal growth in the mature colony (Fu *et al.* 2011). As a result of WCC binding to the *adv-1* promoter, *adv-1* is both light- and clock-regulated (Smith *et al.* 2010). These data suggested that ADV-1 functions to connect downstream developmental processes to circadian clock and light signaling pathways. To elucidate the role of ADV-1 in controlling light signaling and clock output pathways, we used ChIP-seq in wild type (WT) cells, and RNA-seq comparing WT to Δ*adv-1* cells, to identify the ADV-1 direct and indirect target genes. In agreement with the Δ*adv-1* phenotype, ADV-1 direct targets were enriched for genes involved in sexual and asexual development, metabolism, and cell fusion. As predicted, the majority of ADV-1 target genes were either light responsive and/or clock-controlled. Deletion of ADV-1 altered the light responses of target genes in predictable ways, and circadian rhythmicity of certain cell fusion genes was abolished or altered in Δ*adv-1* cells. Taken together, these data reveal that ADV-1 plays a key role in signaling light and temporal information to control *N. crassa* gene expression and development.

## Materials and Methods

### Strains and culture conditions

*N. crassa* strains, including WT 74OR23-1 (FGSC 2489) and Δ*adv-1* (FGSC 11042) were obtained from the Fungal Genetics Stock Center (FGSC, Kansas State University, Manhattan, KS; http://www.fgsc.net). The V5-tagged ADV-1 strain used for ChIP-seq was previously described (Smith *et al.* 2010). Briefly, a V5 tag (14 amino acids, GKPIPNPLLGLDST) was added at the C-terminus of ADV-1, away from the Gal4-like Zn^2^Cys^6^ DNA-binding domain at the N-terminus (aa 50–85) of the 696-aa ADV-1 protein. To examine the phenotype of ΔADV-1 cells, protoperithecia assays were carried out on Westergaard and Mitchell’s synthetic crossing medium containing 0.5% sucrose as a carbon source (Westergaard and Mitchell 1947) (http://www.fgsc.net/neurosporaprotocols/How%20to%20choose%20and%20prepare%20media.pdf). Protoperithecia were scored from cultures grown for 7–8 d in the dark at 20°.

To test if ADV-1 potentially controls its own expression, *adv-1* was overexpressed from the copper-regulatable *tcu-1* promoter at the *his-3* locus. To generate this strain, primers *Pvu*I *adv-1* F and *Pvu*I/*Spe*I *adv-1* R (Supplemental Material, Table S1) were used to amplify the full-length *adv-1* gene from WT *N. crassa* genomic DNA. This DNA fragment was digested with *Pvu*I and then cloned into the corresponding sites of pCR blunt bar::P*_tcu-1_* (Lamb *et al.* 2013), to generate pDBP506. Digestion of pDBP506 with *Spe*I yielded a 4.9 kb bar-P*_tcu-1_-adv-1* fragment that was ligated into *Spe*I cut pBM61 (*his-3* targeting vector) to generate pDBP508. pDBP508 was transformed into *his-3*, *mat a*, and *his-3*, *mat A* strains. His^+^ transformants were selected, and proper integration of the plasmid was validated by PCR, yielding *his-3*^+^::*bar*-P*_tcu-1_-adv-1 mat a* (DBP 1749) and *his-3*^+^::*bar*-P*_tcu-1_-adv-1 mat A* (DBP 1750).

To assay rhythms in ADV-1 protein levels, an ADV-1::LUC translational fusion strain was generated using recombinational cloning in yeast as previously described (Colot *et al.* 2006). To determine the effects of ADV-1 deletion on the rhythmicity of cell fusion genes, cell fusion gene promoter-driven luciferase (LUC) reporter constructs were generated. Five PCR fragments [5′ of *csr*, promoter region of gene, codon optimized *luc* sequence (Gooch *et al.* 2008), 3′ UTR of gene, and 3′ of *csr*] were cotransformed with gapped plasmid (pRS426) digested with *Xho*I and *Bam*HI into yeast strain FY2 (MATα, ura3–52). The primers used, *csr* 5F, *csr* 5R, *csr* 3F, and *csr* 3R, are listed in Table S1. Following recombination, the full-length cassette was amplified and transformed into WT and Δ*adv-1* cells. For the ADV-1::LUC translational fusion, the ADV-1 coding region, minus the stop codon, was amplified using primers NCU07392.Cf and Cr, and ∼1 kb of the 3′ end was amplified using primers NCU07392.3f and 3r (Table S1). The PCR fragments were recombined in yeast with a 10Xgly-*luc-hph* cassette and plasmid pRS426 as described (Colot *et al.* 2006). Following amplification of the fusion cassette, transformation into WT *N. crassa* cells, and homologous recombination at the *adv-1* locus, the strain was crossed to FGSC2489 to generate a homokaryotic strain (DBP1356).

To demonstrate that the ADV-1::V5 tagged protein was functional, P*ham-6*::*luc* was amplified using *csr-1* 5′ flanking region F and *csr-1* 3′ flanking region R primers (Table S1) from DBP1844 (P*ham6*::*luc*, ∆*adv*-1). The PCR product was gel purified using QIAquick (Qiagen, Valencia, CA) and transformed into DBP1054 cells (ADV1::V5). Primary transformants were selected by cyclosporine resistance (5 µg/ml) and positive luciferase activity. Homokaryons were isolated by microconidia filtration (Ebbole and Sachs 1990), and verified by PCR using *csr-1* primers (Table S1).

### Luciferase assays

To assay rhythmicity from transcriptional and translational luciferase fusion constructs, conidia were inoculated into 96-well microtiter plates containing 1× Vogel’s salts, 0.01% glucose, 0.03% arginine, 0.1 M quinic acid, 1.5% agar, and 25 μM firefly luciferin (LUCNA-300; Gold Biotechnology, St. Louis, MO), pH 6.0. Quinic acid was added to the media to increase the amplitude of the luciferase rhythms (Larrondo *et al.* 2015). To synchronize the cells to the same time of day, the plates were incubated in constant light (LL) at 30° for 24 hr, and then transferred to DD 25°. Bioluminescence was measured using a TopCount NXT Microplate Scintillation and Luminescence Counter (PerkinElmer Life Sciences, Boston, MA) with recordings taken every 90 min over 4–5 d. Data were collected using the Import and Analysis (I&A) program (http://www.amillar.org/PEBrown/BRASS/BrassPage.htm). Raw luciferase data were analyzed for period, phase, and amplitude determination using Biological Rhythms Analysis Software System (BRASS) running on BioDARE (Biological Data Repository) (http://www.biodare.ed.ac.uk) (Zielinski *et al.* 2014; Moore *et al.* 2014). The first day of recording was not included in the analyses to avoid potential artifacts caused by transfer of the plates to the TopCount, and the data were detrended to account for a general decrease in amplitude toward the end of the experiments (Zielinski *et al.* 2014; Moore *et al.* 2014).

### RNA, protein, and ChIP analyses

For northern assays, total RNA was extracted from cells ground in liquid nitrogen as described (Bell-Pedersen *et al.* 1996). Transcript levels were detected with [α-^32^P]-UTP-labeled riboprobes (Correa *et al.* 2003). Densitometry was performed using ImageJ software and normalized to ethidium bromide-stained ribosomal RNA. We were unable to detect *prm-1* (NCU09337) mRNA by northern blot analyses. Therefore, quantitative RT-PCR was used to examine *prm-1* mRNA levels as described previously (Wu *et al.* 2014). The *cox-5* gene (NCU05457), which is not regulated by ADV-1, was used as an internal control for normalization. To test ADV-1 autoregulation, allele-specific semiquantitative RT-PCR was performed. Primers that amplified a product common to both the endogenous and ectopic forms of *adv-1* detected “Total *adv-1*” (Table S1). Primers that amplified a 5′ UTR sequence unique to the endogenous *adv-1* gene detected “Native *adv-1*” (Table S1). *ef-1α* expression, which is not copper-responsive, was used as a specificity control. To detect ADV-1::V5 by western blot, ADV-1::V5 conidia (∼1 × 10^7^) were added to Petri dishes containing 25 ml 1× Vogel’s salts, 2% glucose, and 0.5% arginine, pH 6.0, and incubated in LL 25° for 24 hr to prepare mycelial mats. Discs (5 mm in diameter) were cut from the mats and a single disc was inoculated into each flask containing 65 ml of Vogel’s media. The cultures were incubated in LL at 30° for 24 hr with shaking (200 rpm), and then transferred to constant dark (DD) 25° for 24 hr with shaking (200 rpm). The cultures were exposed to light (∼21 μmol photons/m^2^/sec, or ∼1500 lux) for 0, 15, 30, and 60 min. Protein extraction and western blotting were carried out as previously described (Garceau *et al.* 1997) with a 1:5000 dilution of mouse monoclonal anti-V5 antibody (Invitrogen, Carlsbad, CA), or monoclonal anti-actin Clone AC-40 (Sigma-Aldrich, St. Louis, MO) used at a 1:1000 dilution. ADV-1::V5 ChIP was carried out as described (Tamaru *et al.* 2003), with some modifications. Briefly, conidia (∼1 × 10^7^) from ADV-1::V5 cells were inoculated into Petri dishes with either 25 ml of Vogel’s or Bird’s media (Metzenberg 2004) containing 2% glucose, and the cultures were processed as described above for western blotting. Formaldehyde (1%) was added to the cultures following light exposure for 0, 15, 30, and 60 min, and the cultures were incubated for 30 min with shaking. Chromatin was sheared to an average size of 500 bp by sonication (Branson digital sonifier S-250D, microtip probe). The sheared chromatin was immunoprecipitated using 5 μg of mouse monoclonal anti-V5 antibody (Invitrogen, Carlsbad, CA). The immunoprecipitated DNA was extracted with phenol-chloroform, and treated with RNase A for 2 hr at 50°. ChIP DNA was quantified using Picogreen (Invitrogen, Carlsbad, CA) according to manufacturer’s instructions. DNA libraries were prepared from immunoprecipitated chromatin (ChIP) for sequencing (Pomraning *et al.* 2009, 2012). An independent chromatin immunoprecipitation with anti-V5 antibody was carried out using ADV-1::V5 cells to validate ADV-1 binding sites on select genes. PCR primer pairs were designed to amplify specific ADV-1 binding regions using Primer3 (Untergasser *et al.* 2012). PCR primer pairs amplifying the coding region of *cpc-1* was used as a negative control.

### ChIP-seq and data analyses

DNA was end repaired and ligated to adapters (Pomraning *et al.* 2009). Fragments (300–500 bp long) were gel purified and amplified by 21–24 cycles of PCR with Phusion polymerase (Finnzymes Oy, NEB) and Illumina PCR primers (Pomraning *et al.* 2012). Libraries were sequenced on an Illumina GAII and processed with RTA1.8 and CASAVA1.7. Sequencing yields and percent mapped are listed in Table S2.

ChIP-seq reads were sorted by their adapter, and adapter sequences were removed. Quality scores were converted to Sanger format with the MAQ sol2sanger command (Li *et al.* 2008). Fastq files were used as input for BWA (Li and Durbin 2009) and aligned to Assembly 10 of the *N. crassa* genome (http://www.broadinstitute.org/annotation/genome/neurospora/GenomesIndex.html). Assembly 10, rather than Assembly 12, was used in these analyses because of a reversal in orientation of chromosome VII, and an inversion of a single contig in chromosome 6 in Assembly 12. No additional sequences were added in Assembly 12; thus, the gene annotations, upon which all of our conclusions are based, are independent of genome assembly. SAM-formatted alignment files from BWA were converted to bam format, sorted, and indexed with SAMtools (Li *et al.* 2009) for viewing in the gbrowse2 genome browser (Stein *et al.* 2002). Aligned reads are viewable in the gbrowse2 genome browser at http://ascobase.cgrb.oregonstate.edu/cgi-bin/gb2/gbrowse/ncrassa_public/. High throughput sequencing (HTS) data from ChIP- and RNA-seq (below) were submitted to NCBI Sequence Read Archive (Accession SRP034658). Each experiment is designated with a specific HTS number.

Peak calling was performed using a previously described analysis pipeline (Jaini *et al.* 2014; Galagan *et al.* 2013a,b). Briefly, the *pileup* command from the SAMtools suite (Li *et al.* 2009) was used to calculate forward and reverse coverage from the alignment files. The background coverage was modeled with a lognormal distribution. This distribution was used to score each position of the genome to identify enriched positions along the genome. Positions with a *P* value of 0.01 or lower are called enriched. Only regions of enrichment >100 bp were included for further analysis. Since TF binding produces a strand-specific shift in binding in ChIP-seq data (Valouev *et al.* 2008), we applied a cross-correlation filter to further filter for only those enriched regions with at least a 60-bp shift between forward and reverse peaks. Consensus motifs were identified using MEME, where the *P* value represents the likelihood of a given profile scoring well against a randomly generated sequence, and accounting for the length of the sequence (Machanick and Bailey 2011; Bailey *et al.* 2006).

### RNA-seq and data analysis

RNA extraction from WT and Δ*adv-1* cells, mRNA enrichment, cDNA preparation, and library preparation for sequencing was performed as previously described (Wu *et al.* 2014). Sequencing was carried out on a HiSeq2000 and analyzed with RTA 1.13.48 and CASAVA v1.8.2. Sequencing yields are listed in Table S2. Following adapter sequence removal, reads were mapped to *N. crassa* Assembly 10 with Tophat using options -a 5 -m 1 -i 30 -I 2000 -p 5–library-type fr-unstranded. Aligned reads can be viewed at http://ascobase.cgrb.oregonstate.edu/cgi-bin/gb2/gbrowse/ncrassa_public/. Tophat output was sorted and indexed with SAMtools (Li *et al.* 2009) and used as input for cuffdiff analysis to identify differentially expressed genes between WT and Δ*adv-1* strains. RNA-seq across a light-induction time course in WT *N. crassa* was previously described (Wu *et al.* 2014), and the data are available under GEO series accession GSE53534. Cuffdiff (Cufflinks Suite Version 2.0) was run using two WT replicates and two Δ*adv-1* replicates (Table S2) using the Broad version 7 reference transcriptome (http://www.broadinstitute.org/annotation/genome/neurosporaMultiDownloads.html).

### Functional enrichment analysis

The Gene identifiers assigned to different Gene Ontology (GO) terms were mapped using the GO Term Finder tool (http://go.princeton.edu/cgi-bin/GOTermFinder) (Boyle *et al.* 2004) with an *N. crassa* GO terms association file (go_for_nc12.tsv downloaded from http://www.broadinstitute.org/annotation/genome/neurospora/Downloads.html). Functional enrichment for direct and indirect ADV-1 targets was determined using the FunCat tool (FunCatDb) (Ruepp *et al.* 2004). The statistical significance of enrichment of gene groups in functional categories relative to the whole genome is based on hyper-geometric distribution. Heat maps were generated with log_2_ RPKM (reads per kilobase of exon per million) values using Cytoscape clusterMaker plugin (Morris *et al.* 2011).

### Statistical analysis

Nonlinear regression to fit time course data to a sine wave (fitting period, phase, and amplitude) or a line (fitting slope and intercept) as well as Akaike’s information criteria to compare the fit of each data set to the two equations were carried out using the Prism software package (GraphPad Software Inc., San Diego, CA). The *P* values reflect the probability that a sine wave fits the data better than a straight line, and is considered to be rhythmic. Statistical analyses were performed using the Student’s *t*-test, where *P* < 0.05 was considered to be statistically significant.

### Data availability

Strains are available upon request, or from the FGSC (http://www.fgsc.net). Table S1 contains primer information. Table S2 contains a summary of the sequencing data and unique identifier information. Table S3 contains ADV-1 ChIP-seq target genes. Table S4 contains GO analyses of the ADV-1 target genes from ChIP-seq. Table S5 contains RNA-seq data from WT and ADV-1 deletion cells. Table S6 contains a list of genes that are both direct target of ADV-1 and are differentially regulated in WT *vs.* ADV-1 deletion cells. Table S7 contains GO and FunCat analyses of genes differentially regulated by ADV-1. Table S8 contains a comparison of ADV-1 target genes and differentially regulated genes to light- and clock-controlled genes. Table S9 contains GO analyses of ADV-1 target genes that are clock-controlled. Table S10 contains genes that are differentially regulated by ADV-1 and are predicted to be clock-controlled. Gene expression data are available at NCBI Sequence Read Archive with accession SRP034658 and are labeled with each specific HTS number indicated in Table S2.

## Results

### ADV-1 protein levels are clock-controlled

The promoter of *adv-1* is bound by WCC, and *adv-1* mRNA levels are transiently induced by light in WT, but not in Δ*wc-1* cells (Smith *et al.* 2010). Consistent with WCC binding and activating the *adv-1* promoter, *adv-1* mRNA levels are low in Δ*wc-1* cells as compared to WT cells, and *adv-1* mRNA accumulates rhythmically in WT, but not in Δ*wc-1* cells (Smith *et al.* 2010). To confirm that the rhythm in *adv-1* mRNA levels leads to rhythms in protein abundance, we created a translational fusion between ADV-1 and codon-modified luciferase (LUC) (Gooch *et al.* 2008). As expected, LUC activity was robustly rhythmic in constant darkness (DD) with a period of 20.3 ± 0.3 hr, and with peak levels during the late subjective day (DD20) (Figure S1 and File S1), slightly lagging the DD16 peak previously observed in *adv-1* mRNA levels (Smith *et al.* 2010). ADV-1-LUC rhythms were abolished in Δ*frq* cells, demonstrating that the rhythm is dependent on a functional FRQ/WCC oscillator (Figure S1). These data prompted further investigation of the role of ADV-1 in light signal transduction pathways, and in circadian clock output pathways, that regulate downstream *ccgs*.

### ADV-1 direct targets, identified by ChIP-seq, are highly enriched for genes involved in metabolism, development, and cell fusion

**Figure 1:**
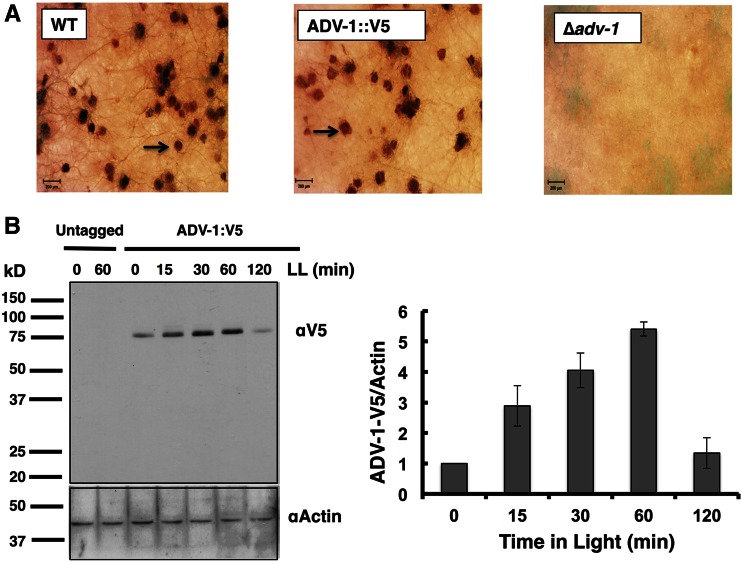
ADV-1::V5 is functional. (A) WT, ∆*adv-1*, and ADV-1::V5 strains were grown on synthetic crossing medium (SCM) to initiate female sexual development, and photographed (25×; scale bar, 200 µm). Protoperithecia (arrow) were observed in WT (left), and ADV-1::V5 (middle), but not in ∆*adv-1* (right) cells. (B) A representative western blot of ADV-1::V5 protein from untagged and ADV-1::V5-tagged cells that were exposed to light for the indicated times (minute) and probed with anti (α)-V5 antibody (top). The same membrane was probed with α-actin antibody to show even protein loading. ADV-1::V5 levels are plotted on the right (± SD, *n* = 2).

To identify downstream genes controlled by ADV-1, we carried out ChIP-seq in a strain containing C-terminal V5 epitope-tagged ADV-1 at the native locus (ADV-1::V5). WT and ADV-1::V5 cells, but not Δ*adv-1* cells, produced normal protoperithecia, demonstrating that the tagged protein was functional for developmental processes (Figure 1A). ADV-1::V5 protein (∼79 kDa) was specifically detected in western blots using anti-V5 antibody. ADV-1::V5 protein was observed in DD, and the levels were increased following 15, 30, and 60 min of light treatment (Figure 1B). Therefore, ADV-1::V5 ChIP-seq using anti-V5 antibody was carried out on cells harvested after growth in the dark (LL0), and after light treatment (LL) for 15, 30, and 60 min to induce the levels and genome-wide binding of ADV-1::V5. Sequence reads obtained for the ChIP samples, and the percent mapped to the *N. crassa* genome are shown in Table S2.

**Figure 2:**
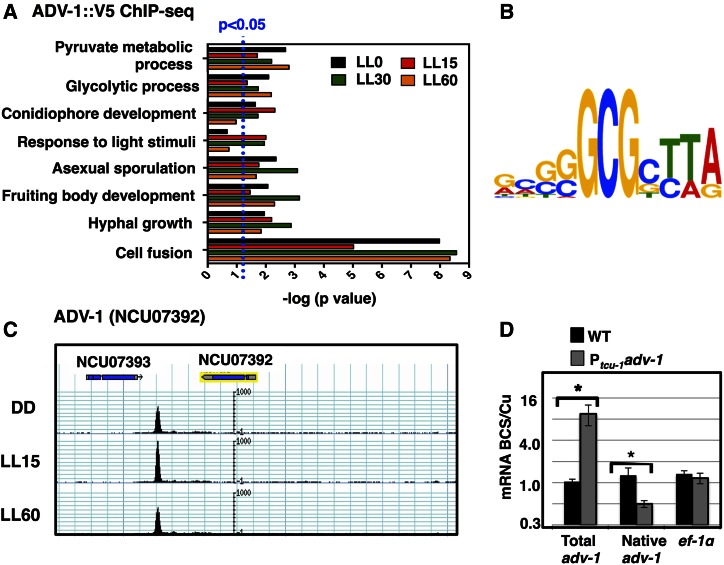
ADV-1 direct targets are enriched for genes involved in light responses, development, and metabolism, and ADV-1 potentially negatively regulates its own expression. (A) GO analysis of predicted ADV-1 direct target genes identified by ChIP-seq from cultures grown in the dark (LL0) and in LL for 15, 30, and 60 min. The GO terms (*y*-axis) for significantly enriched categories are shown (*P* ≤ 0.05; indicated by the dotted blue line) at one or more time points (*x*-axis). For visualization, the *P* values are plotted as the −log. (B) Analyses of ADV-1 binding sites in the 30 most significant peaks identified by ChIP-seq revealed an ADV-1 consensus-binding site. The relative height of each nucleotide (shown in the 5′ to 3′ direction) reflects the degree of sequence conservation in the ADV-1 consensus-binding site. (C) ChIP-seq track showing ADV-1::V5 binding downstream of the *adv-1* coding region from cultures harvested after growth in the dark (DD) or following a 15 (LL15) or 60 (LL60) min light treatment. (D) Plot of the relative abundance under inducing (250 µM BCS) *vs.* repressing conditions (250 µM CuSO_4_) of the indicated messages in WT (black boxes) and P*_tcu-1_ adv-1* (gray boxes) strains (*n* = 3). The asterisks represent statistical significance by a Student’s *t*-test (* *P* < 0.002).

Using a stringent statistical cutoff value for peak calling [false discovery rate (FDR) <0.01] (Jaini *et al.* 2014; Galagan *et al.* 2013a), and stipulating that the ADV-1 binding site occurs within 5 kb upstream of the transcription start site, 3 kb downstream of the transcription stop site, or within the coding region, 320, 636, 458, and 308 ADV-1-binding sites were identified in the genome from the 0, 15, 30, and 60 min light-treated ChIP samples, respectively (Table S3). The average intergenic distance in *N. crassa* is ∼2 kb (Galagan *et al.* 2003); however, we used longer upstream and downstream cutoffs to identify ADV-1 binding sites near genes, with the cutoffs based on functional WC-2 binding sites located up to 4 kb upstream of a gene start site (Smith *et al.* 2010), and based on identification of an ADV-1 binding site located ∼3 kb downstream of the 3′ end of *adv-1* mRNA (Figure 2C). The number of genes assigned to ADV-1-binding sites was greater than the number of binding-site peaks due to the presence of binding-site peaks between two divergently transcribed genes (Table S3). This ChIP-seq gene set (491 genes at LL0, 922 genes at LL15, 665 genes at LL30, and 456 genes at LL60) was used for all subsequent analyses. Combining genes from all time points revealed that ADV-1 potentially controls the expression of 1029 unique gene targets (Table S3), representing ∼10% of the predicted 9730 *N. crassa* genes. Several of the predicted ADV-1 target genes are also predicted targets of the WCC, and/or of two downstream TFs that are direct targets of the WCC, CSP-1, and SUB-1, suggesting combinatorial control for these genes (Smith *et al.* 2010; Sancar *et al.* 2011, 2015a) (Table S3). The distribution of ADV-1 binding peaks for each time point, based on their positions relative to the transcriptional start site from the annotated genes in the *N. crassa* genome (http://www.broad.mit.edu/annotation/genome/neurospora/), were examined (Figure S2A). In all four dark or light conditions, ∼67% of the ADV-1 binding sites were present upstream of the transcriptional start site, ∼31% were downstream of the coding sequence, and the remaining binding sites (∼2%) were located in gene coding regions.

The expression of ADV-1::V5 (Figure 1B), and the presence of ADV-1::V5-bound peaks, in the dark suggested that light stimulation is not necessary for ADV-1::V5 to be bound at some genomic locations. The number of ADV-1 binding sites increased ∼twofold after a 15-min light treatment (Table S3), consistent with the observed increase in ADV-1::V5 protein levels compared to dark-grown cultures (Figure 1B). Interestingly, a decrease in the number of binding sites was observed in the 30- and 60-min light-treated samples relative to the 15-min sample, despite higher levels of ADV-1::V5 protein in these cells relative to cells exposed to light for 15 min (Figure 1B), suggesting that longer light treatments reduce ADV-1::V5 binding to its target sequences. About 90% of the binding sites observed from cultures given 30 min of light overlapped with those observed with cells treated with 15 min of light, and about 60% of the binding sites observed from cultures given 60 min of light overlapped with those observed with cells treated with 30 min of light (Figure S2B and Table S3).

GO analysis (Boyle *et al.* 2004) was used to identify the functional categories of genes bound by ADV-1 (Figure 2A and Table S4). Genes involved in metabolism, asexual and sexual development, response to light, hyphal growth, and cell fusion are ADV-1 targets (Figure 2A). The GO categories enriched were similar at all time points, with the exception of conidiophore development (at 60 min) and response to light stimuli (at 0 and 60 min). ADV-1 deletion strains are defective in the development of protoperithecia (Figure 1; Colot *et al.* 2006); thus, it was not surprising to find an enrichment for ADV-1 target genes involved in fruiting body development. In addition, genes involved in cell fusion were highly enriched, consistent with the absence of CATs in Δ*adv-1* cells; CAT formation facilitates cell fusion (Fu *et al.* 2011; Leeder *et al.* 2013; Read *et al.* 2012), and ADV-1 functions to regulate the expression of essential cell fusion genes. Thus, this genome-wide approach identified expected targets and new pathways, including metabolism, that ADV-1 is predicted to be regulating.

A consensus ADV-1 binding motif was derived from the 30 most statistically significant ADV-1 ChIP peaks common to all growth conditions using the MEME motif discovery tool (Bailey *et al.* 2006; Machanick and Bailey 2011) (Figure 2B). This motif, consisting of GCGCTTA, was enriched among all ADV-1 binding sites in the light, but only for the top 30 peaks in the dark, suggesting that for some binding sites, ADV-1 potentially recognizes different consensus sequences in the dark *vs.* the light (Figure S3), possibly through partner interactions.

A replicate ADV-1::V5 ChIP was used to validate the ChIP-seq results. ChIP-PCR was used on the replicate to examine binding at 10 regions that were randomly selected from among the predicted binding regions in the ChIP-seq studies (Figure S4). In all cases, enrichment shown by ChIP-PCR validated the ADV-1 ChIP-seq results (Figure S5). For example, for NCU04847, two distinct ADV-1-bound peaks are located upstream of the gene (Figure S5); PCR validation confirmed ADV-1 binding at both sites (Figure S4). These experiments also validated binding of ADV-1 to sequences at the 3′ end of the converging *adv-1* and NCU07393 genes (Figure 2C). Binding of ADV-1 to its own 3′ control region suggested the possibility of feedback regulation. No other ADV-1 binding sites were identified within 10 kb upstream or downstream of the *adv-1* gene (Figure 2C).

To begin to test if ADV-1 controls its own expression, we overexpressed *adv-1* from a copper-controlled promoter (P*_tcu-1_*) integrated at the *his-3* locus and examined the effect on expression from the endogenous *adv-1* locus. P*_tcu-1_*-driven expression is activated by BCS (bathocuproinedisulfonic acid), a copper chelator, and repressed by high copper concentrations (Lamb *et al.* 2013). Expression of *adv-1* or control gene *ef-1α* was not dependent on the BCS/Cu concentrations in a WT cell as expected (Figure 2D). In a strain carrying ectopic P*_tcu-1_-adv-1*, the total levels of *adv-1* mRNA were increased by BCS as expected, while the expression of the *ef-1α* was unaffected. However, overexpression of *adv-1* integrated at *his-3* led to a significant decrease in expression from the native *adv-1* locus, suggesting that ADV-1 has the potential for negative autoregulation.

Additional validation of the ADV-1::V5 ChIP-seq data was accomplished by carrying out ADV-1::V5 ChIP-seq on cells grown in Bird’s medium (Table S2) instead of Vogel’s medium (which was used for the light-induction studies described above). A comparison between the two media was of interest because while Vogel’s medium is routinely used for *N. crassa* growth in laboratories, Bird’s medium was recommended for use in genomic studies owing to enhanced pH stability and the inclusion of a single nitrogen source, when compared to Vogel’s medium (Metzenberg 2004). We found that the correlation between the ADV-1 peaks determined by ChIP-seq from cells grown in both Vogel’s and Bird’s media was high for all conditions (*R*^2^ ≥ 0.87) (Figure S6), further validating the original ChIP-seq data and demonstrating that there are no major differences in ADV-1 function in the two media.

### RNA-seq reveals complex regulation of ADV-1 target genes

To determine how ADV-1 affects gene expression, mRNA was isolated from WT and Δ*adv-1* cells grown in the dark, and following light treatment for 15, 30, and 60 min and used for RNA-seq. These experiments were carried out using two independent biological replicates, and statistical analyses of the differentially expressed genes were performed using the Cufflinks tool, Cuffdiff (Trapnell *et al.* 2013). Sequencing data obtained for each replicate are summarized in Table S2, and the raw data are presented in Table S5. Comparison between WT and Δ*adv-1* cells showed that, among 9726 predicted protein coding genes, there were ≥twofold differences in expression levels for 1472 mRNAs (15%) in the dark (LL0), 1770 mRNAs (18%) at LL15, and 2177 mRNAs (22%) at LL60 (Table S5). To increase the stringency, we applied a *q*-value, representing the minimum false discovery rate (Storey and Tibshirani 2003), of ≤0.2 to the data which matched the stringency set for examining light responses in *N. crassa* (Wu *et al.* 2014); 305 mRNAs (3%) at LL0, 418 mRNAs (4%) at LL15, and 494 mRNAs (5%) at LL60 met both criteria (Table S5). While there were specific mRNAs that showed changes in levels that were dependent on the growth conditions of the cell, the overall number of differentially expressed genes was similar in the dark- and light-treated cells. Combining all time points, 804 unique genes had mRNA levels that changed ≥twofold with a *q*-value of ≤0.2 in WT compared to Δ*adv-1* cells in the dark and following light treatment (Table S5). This gene set represents ∼8% of the 9730 expressed genes, and was used for all subsequent analyses. Applying a more stringent cutoff, *q*-value of ≤0.1, to the unique gene set reduced the number of differentially expressed genes to 514 (Table S5), but did not change our overall conclusions.

**Figure 3:**
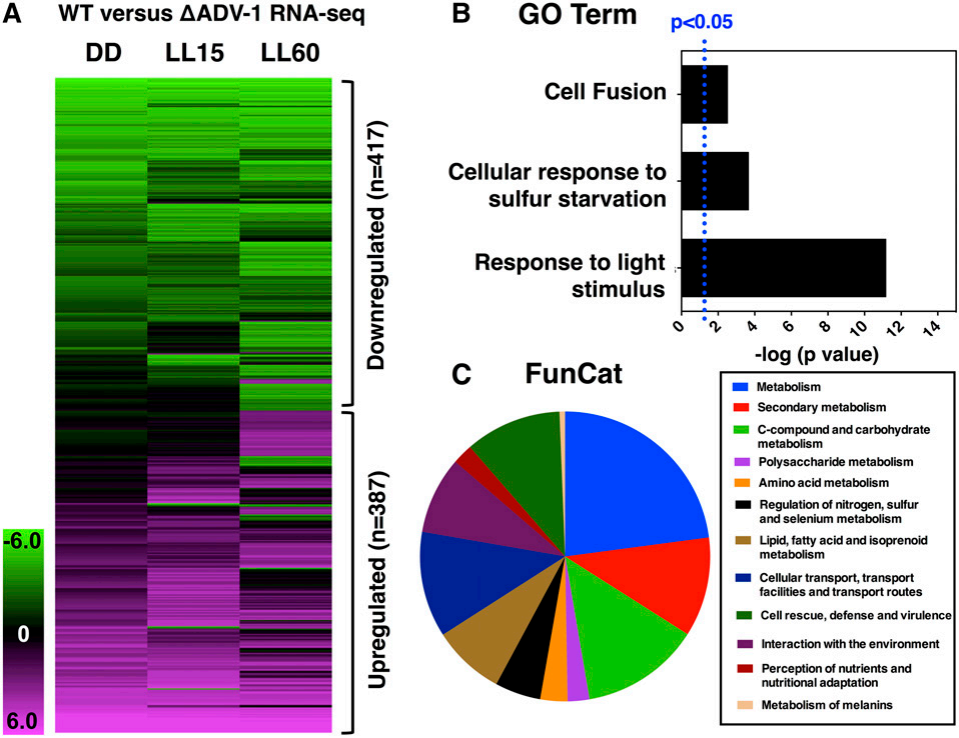
RNA-seq in WT *vs.* ∆*adv-1* cells identified differentially expressed mRNAs. (A) Heat map of 804 unique genes that are differentially expressed between WT and ∆*adv-1* cells following 0, 15, and 60 min of light exposure. The genes were considered differentially expressed if they differed in at least one of the conditions by ≥twofold and *q*-value ≤ 0.2. The columns show the growth conditions, and the rows represent individual target genes. Green represents reduced mRNA levels in ∆*adv-1* cells (417 genes), and pink represents increased mRNA levels in ∆*adv-1* cells (387 genes), as compared to WT cells. (B) GO analysis of the 804 genes regulated by ADV-1. The most highly significant enriched GO terms compared to all categories are shown (*P* ≤ 0.05). The plot is labeled as described in Figure 2. (C) Functional Category (FunCat) enrichment analysis (*P* ≤ 0.05) of the 804 genes regulated by ADV-1. The distributions of the top categories are shown as a pie chart.

Figure 3A shows hierarchical clustering of the 804 unique differentially expressed genes. Similar numbers of genes were activated or repressed by ADV-1, suggesting that ADV-1 directs both positive and negative trajectories of gene expression. A comparison between the ADV-1 ChIP-seq targets and the 804 ADV-1-regulated genes identified 139 genes that are directly controlled by ADV-1 (Table S6). Of these 139 genes, 110 (80%) were activated by ADV-1, whereas 29 genes (20%) were repressed by ADV-1, indicating that ADV-1 primarily functions as a transcriptional activator of its direct targets. Furthermore, the poor overlap between the differentially expressed genes and the direct targets of ADV-1 revealed that binding does not necessarily equate with a change in mRNA levels. This may be due to binding and compensation by other TFs, such as WCC, SUB-1, and CSP-1 (Sancar *et al.* 2011, 2015a; Smith *et al.* 2010) (Table S3), and/or may reflect that regulatory mechanisms other than light are also important for controlling the expression of these ADV-1 targets.

GO analyses were used to determine functional categories of the 804 unique genes differentially controlled by ADV-1 (Figure 3B and Table S7). As expected, enrichment for light-responsive genes was observed. Genes involved in cell fusion were also enriched, consistent with the ADV-1 ChIP-seq data. In addition an enrichment was observed for genes involved in sulfur metabolism, based on differential expression of genes involved in sulfur assimilation, including NCU04433 (*cys-14*) encoding sulfate permease (Jarai and Marzluf 1991), and NCU06041 (*ars-1*) encoding aryl sulfatase-1 (Marzluf 1993) (Table S7). For a more detailed view of the pathways potentially controlled by ADV-1 the same gene set was analyzed using FunCat (Ruepp *et al.* 2004), where enrichment for specific metabolic pathways influenced by ADV-1, including nitrogen, sulfur, amino acid and carbohydrate metabolism, was observed (Figure 3C and Table S7). Furthermore, genes involved in transport and environmental signaling were enriched, as well as genes involved in melanin metabolism. Melanin helps protect fungal cells, particularly cells produced during sexual development in *N. crassa*, from UV light damage (Butler and Day 1998), further supporting a link between ADV-1 and the sexual cycle. GO and FunCat analyses of the list of differentially regulated genes with a ≥twofold change and a *q*-value of ≤0.1 in WT compared to Δ*adv-1* cells yielded similar results (Table S7)

### ADV-1 regulates light-responsive genes and clock-controlled genes

**Figure 4:**
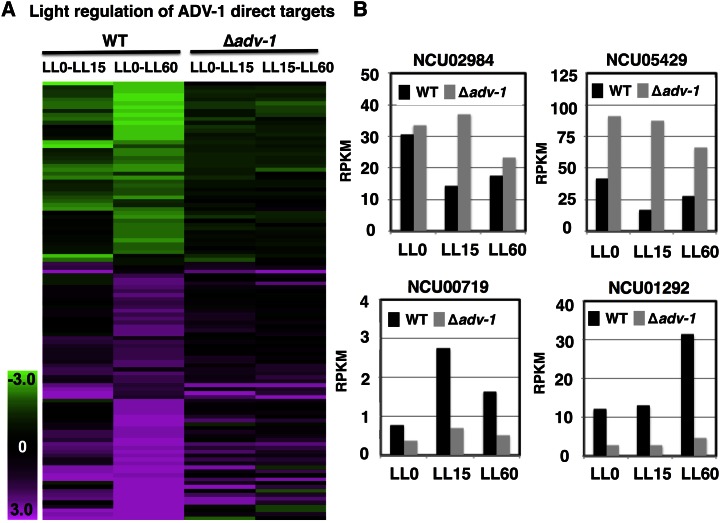
Heat map of 112 direct targets of ADV-1 (rows) whose mRNA levels are light-regulated. The levels of mRNA from cells grown in the dark, or exposed to light for 15 and 60 min, in WT and Δ*adv-1* cells are shown where green represents downregulation and pink represents upregulation in the light *vs.* dark conditions. (B) The mRNA levels (average RPKM values) of representative ADV-1 direct target genes from RNA-seq analyses in WT cells (black bars) and Δ*adv-1* cells (gray bar) grown in the dark (LL0) and harvested after light exposure for 15 (LL15) and 60 (LL60) min (*n* = 2, fold change ≥2×, *q* ≤0.2).

Comparisons between the direct gene targets of ADV-1 to *N. crassa* light-regulated genes identified by RNA-seq (Wu *et al.* 2014) revealed that ADV-1 has the potential to transduce light signals to some (∼30%), but not all, of its direct target genes (Table S8). Among the genes that are direct targets of ADV-1 and are light-regulated, about half were activated by light and half were repressed by light (Figure 4A), and deletion of *adv-1* substantially altered the light response as predicted. For example, light repressed NCU02984 and NCU05429 mRNA levels in WT cells, but the mRNA levels were high in the light in Δ*adv-1* cells (Figure 4B). Light-activated expression of NCU00719 and NCU01292 in WT cells, but the mRNA levels were low in the light in Δ*adv-1* cells.

**Figure 5:**
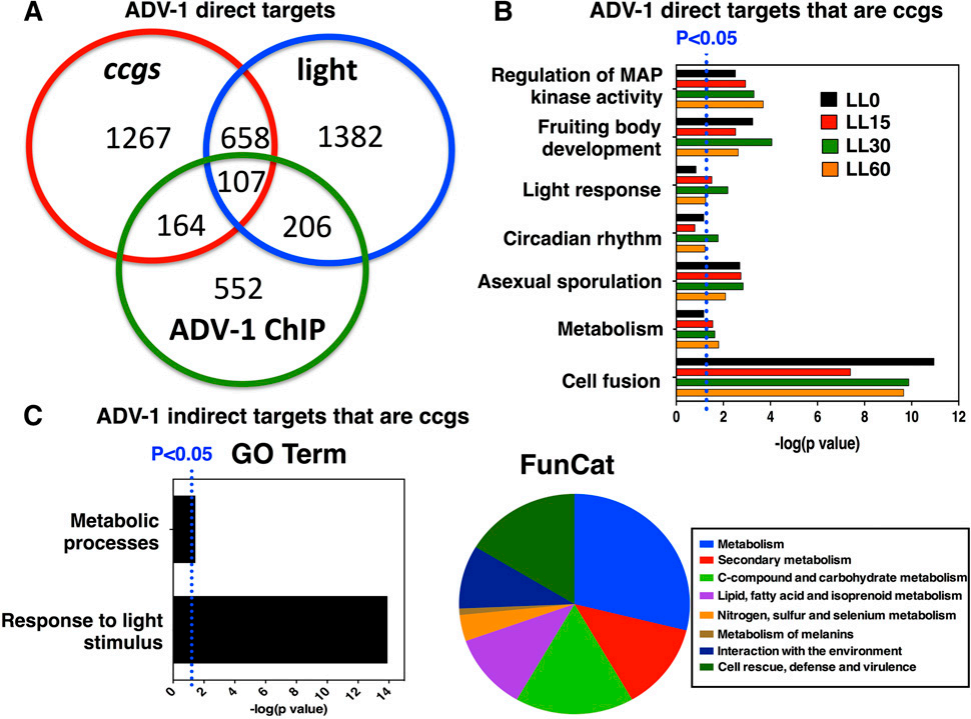
ADV-1 downstream targets identified by ChIP-seq and RNA-seq are enriched for *ccgs*. (A) Venn diagram comparing predicted ADV-1 direct target genes identified by ChIP-seq to light-regulated genes and predicted and known *ccgs*. (B) GO and FunCat analyses of clock-controlled and ADV-1-bound genes. (C) The 268 genes that are both indirect targets of ADV-1 based on RNA-seq and are predicted or known *ccgs* (Table S10) were analyzed using GO and FunCat and the most highly significant enriched categories are represented (*P* ≤ 0.05). The plots are as described in Figure 2.

The rhythmic accumulation of ADV-1 levels led us to predict that many of the ADV-1 targets would be under clock control. To identify rhythmic targets of ADV-1, a comparison between the ADV-1 direct targets at each time point to the unique 2196 *ccgs* identified from rhythmic microarrays (Dong *et al.* 2008; Lewis *et al.* 2002; Nowrousian *et al.* 2003) and RNA-seq (Hurley *et al.* 2014; Sancar *et al.* 2015b) was made. Approximately one-third of all potential direct ADV-1 targets are predicted *ccgs* (271 unique *ccgs*) (Figure 5A and Table S8). Comparing the set of predicted direct ADV-1 targets to the sets of light-responsive genes and *ccgs* showed that 10% are both clock- and light-regulated, and 53% are neither clock- nor light-controlled. In addition, 20% of the direct ADV-1 targets were light- but not clock-controlled, and 16% were clock- but not light-regulated (Figure 5A and Table S8). Thus, many, but not all, of the direct ADV-1 target genes are *ccgs*, and not all light-regulated genes are clock-controlled.

GO analysis was accomplished to determine the functional categories enriched for *ccgs* directly bound by ADV-1. The enriched categories included fruiting body development, circadian rhythms, light responses, metabolic processes, and cell fusion (Figure 5B and Table S9), similar to the enrichment observed for all ADV-1 direct targets (Figure 2B).

To determine if temporal information is transduced downstream of the ADV-1 direct targets, we examined if any of the 665 genes that are differentially regulated by ADV-1, but are not directly bound by ADV-1 (indirect targets), are predicted *ccgs* (Table S8). We identified 268 (40%) genes as predicted *ccgs*, only two of which are also direct targets of the WCC (Smith *et al.* 2010), and about half of which are activated and half are repressed by ADV-1 based on the RNA-seq data (Table S10). Pathway analyses of these *ccgs* (GO and FunCat) revealed enrichment for pathways (Figure 5C) that were similar to the full set of differentially expressed genes (Figure 3, B and C). Together, these data indicated that ADV-1 functions within a circadian output pathway, potentially controlling rhythmicity for both direct and indirect gene targets involved in development, environmental sensing, metabolism, and cell fusion. Clock control of development, environmental sensing, and metabolism are well established (Brown 2014; Summa and Turek 2014; Goldsmith and Bell-Pedersen 2013; Bass and Takahashi 2010). However, the data indicating that the clock regulates aspects of cell fusion were unexpected.

### ADV-1 transduces rhythmic signals from the clock to control rhythms in cell fusion gene mRNA accumulation

**Figure 6:**
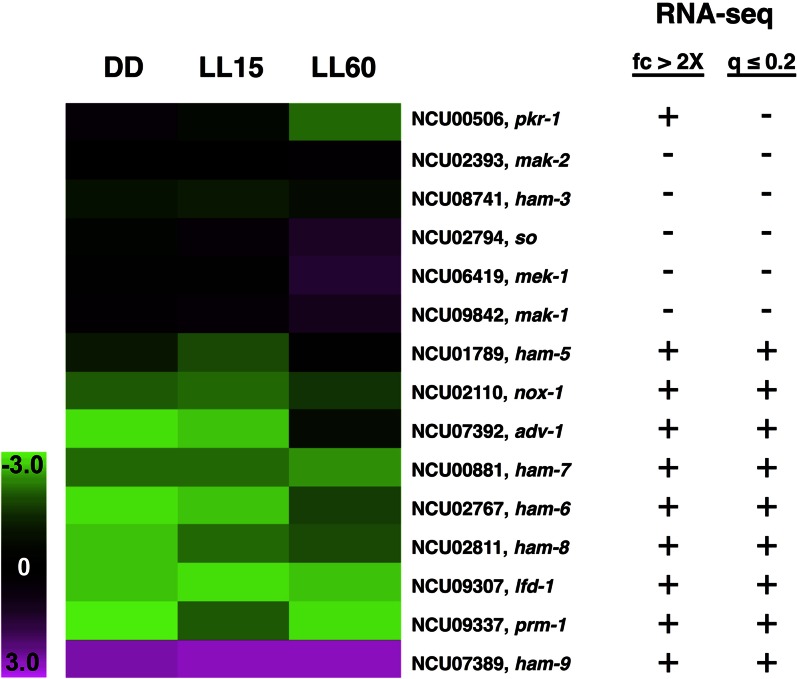
ADV-1 regulates genes involved in cell fusion. Heat map of 15 cell fusion genes identified as direct ADV-1 targets showing the fold change in mRNA levels in WT as compared to ∆*adv-1* cells following 0, 15, and 60 min of light exposure. Green indicates reduced mRNA levels in ∆*adv-1* cells and pink represents increased mRNA expression in ∆*adv-1* cells, as compared to WT cells. The columns show the growth conditions, and the rows represent individual cell fusion genes in the heat map. Indicated on the right is whether (+) or not (−) the mRNAs changed twofold or more (fc ≥ 2), and had a *q* ≤ 0.2 in the RNA-seq analyses.

Deletion of cell fusion genes results in delays in, or elimination of, protoperithecia development, similar to the phenotype of Δ*adv-1* cells (Fu *et al.* 2011; Lichius and Lord 2014). Importantly, both Δ*adv-1* cells, and many cell fusion gene deletion strains, lack CATs (Leeder *et al.* 2013; Fleissner *et al.* 2009b; Fu *et al.* 2011), which home toward each other to facilitate cell fusion. ADV-1 binding peaks were identified in the promoters of 15 cell fusion genes (Figure S5 and Table S3), and RNA-seq revealed that 10 cell fusion genes showed at least a twofold change in mRNA levels in cells deleted for *adv-1* as compared to WT cells, and nine of these met the more stringent criteria (*q*-value ≤0.2) (Figure 6). Of the 10 cell fusion genes with differential expression in WT *vs.* Δ*adv-1* cells, all were found by ChIP-seq to be direct targets of ADV-1. The ADV-1-targeted cell fusion genes are predicted to function in all three steps of cell fusion, including chemoattraction, adhesion, and membrane fusion (Read *et al.* 2012). While changes in gene expression are apparent in the heat map for all of the cell fusion genes, some of the genes did not make the stringent statistical RNA-seq cutoff for significance. This may reflect low-level expression of these genes under the growth conditions used in RNA-seq. In any case, northern blot and qRT-PCR analyses confirmed ADV-1 regulation for three cell fusion genes [*ham-6* (NCU02767), *ham-9* (NCU07389), and *prm-1* (NCU09337)] that had a twofold change in mRNA levels (Figure S7). Interestingly, the expression profiles revealed both positive and negative regulation by ADV-1 for the cell fusion genes (Figure 6 and Figure S7). For example, low mRNA levels were observed for *ham-6* and *prm-1*, and high mRNA levels were found for *ham-9*, at all time points in the absence of ADV-1 (Figure S7). Some cell fusion gene mRNA levels were higher (*e.g.*, *so* and *mek-1*) or lower (*e.g.*, *pkr1* and *ham-5*) only in the light-treated samples (Figure 6), although none of the cell fusion genes were previously identified as being light-responsive (Wu *et al.* 2014).

**Figure 7:**
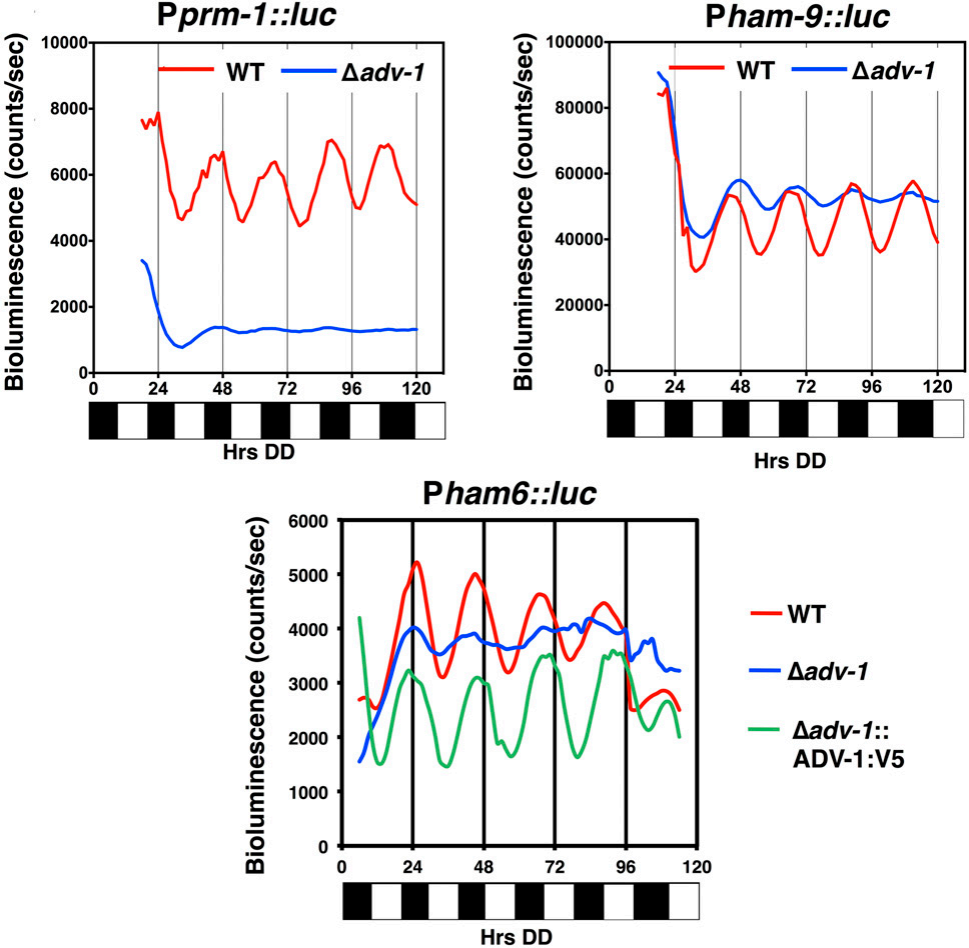
ADV-1 affects the levels and rhythmic expression of cell fusion genes. Representative bioluminescence records (*n* = 12) of WT (red), Δ*adv-1* (blue), and *adv-1*::V5 complemented Δ*adv-1* (green) strains, containing the promoters of the indicated cell fusion genes driving luciferase expression. Bioluminescence levels are indicated on the *y*-axis. The time of recording in constant darkness (DD) is shown on the *x*-axis (hours). The bar graph below each plot represents subjective day (white) or subjective night (black) in DD.

To determine if ADV-1 relays time of day information to control cell fusion gene targets, we asked if the cell fusion genes are *ccgs*, and if so, whether their rhythms depended upon ADV-1. First, promoter::LUC fusion reporters were generated for *prm-1* and *ham-6*, two cell fusion genes that are activated by ADV-1, and for *ham-9*, which is repressed by ADV-1. The expectation was that genes that are activated by ADV-1 would have reporter mRNA levels that peak during, or slightly after, the peak of ADV-1 protein levels in the late subjective day/early night, and genes that are repressed by ADV-1 would peak in antiphase, during the late subjective night/early morning. The promoters of all three genes were shown to drive robust rhythms in LUC expression, with all of the genes peaking during the subjective early night (DD22-DD24) (Figure 7). Thus, the levels of activated genes peaked at the predicted time of day, while the levels of the repressed gene, *ham*-9, did not. These data indicated that ADV-1 might not be sufficient to regulate the phase of rhythmic *ham-9* gene expression. Furthermore, no correlation was observed between the time of peak *ccg* mRNA levels and activation or repression of gene expression by ADV-1 (Figure S8).

To test if additional factors are required for rhythmicity of the cell fusion genes, we assayed LUC rhythms from the same constructs in Δ*adv-1* cells. The rhythms in *prm-1* and *ham-6* promoter activities were severely dampened in the mutant as compared to WT (Figure 7), indicating that ADV-1 is necessary to drive their mRNA rhythms. For *ham-9*, the overall levels of mRNA were increased in ΔADV-1 cells compared to WT cells; however, mRNA rhythms with low amplitudes persisted in ΔADV-1 cells (Figure 7). These data are consistent with ADV-1 being necessary to transduce temporal information to at least some of its direct target genes (*prm-1* and *ham-6*), but that additional factors are needed to control rhythms in the expression of others (*ham-9*). Finally, complementation of the loss of *ham-6* rhythmicity in ΔADV-1 cells by transformation of *adv-1*::V5 provided independent confirmation on the functionality of the ADV-1::V5 fusion protein used for ChIP-seq (Figure 7), and demonstrated that the loss of *ham-6* rhythmicity was due to deletion of ADV-1.

## Discussion

To investigate how light and the circadian clock control gene expression, we used ChIP-seq and RNA-seq to identify the direct and indirect gene targets of the *N. crassa* TF ADV-1. The ADV-1 target genes identified were compared with *N. crassa* genes identified in other genome-wide studies of light-regulated genes and *ccgs* (Wu *et al.* 2014; Hurley *et al.* 2014; Lewis *et al.* 2002; Dong *et al.* 2008; Sancar *et al.* 2011, 2015b). Together, these data sets provide a rich resource to unravel the role of ADV-1 in transducing light and temporal information to downstream targets, and to connect these gene targets to physiological processes. For example, this work led to the discovery of a role for ADV-1 in regulating temporal expression of cell fusion genes.

Consistent with a major function for ADV-1 in controlling gene expression, ∼12% of all predicted *N. crassa* genes had ADV-1 binding sites within 5 kb upstream to 3 kb downstream, or within, the predicted open reading frame. Surprisingly, significantly fewer genes (4%) met the stringent criteria (≥twofold change and *q* ≤ 0.2) for being differentially regulated in Δ*adv-1* cells based on RNA-seq; the overlap between ChIP-seq and RNA-seq data sets was 5%. Relaxing the RNA-seq stringency (any gene with ≥twofold change) significantly increased the overlap between the two data sets to 42%. In either case, not all genes showed differential expression demonstrating that ADV-1 binding to its target sequence is not always sufficient to activate or repress transcription. ChIP-seq analyses of the WCC (Smith *et al.* 2010), and several other TFs that are direct targets of the WCC (R. Dekhang, *et al.*, unpublished results), including CSP-1 (Sancar *et al.* 2011) and SUB-1 (Sancar *et al.* 2015a), showed binding of these TFs to many of the same ADV-1 target gene promoters (Table S3). Thus, binding of additional TFs to ADV-1 target sites may compensate for the loss of ADV-1. Furthermore, while ADV-1, in most instances, functions as an activator of transcription of its direct targets, it can also act as a repressor. No obvious difference in the binding sequences of repressed *vs.* activated genes was identified. Therefore, it is likely that repression results from coordinate TF activity. Genome-wide studies in *Drosophila* (Orian *et al.* 2005) and human cell lines (Cusanovich *et al.* 2014) found similar discrepancies in which the number of TF binding sites far exceeded the number of genes that are differentially regulated, consistent with coordinate TF activity, and/or TF complexes, controlling gene expression.

ADV-1 binding to genomic loci increased twofold in cells given a 15-min light treatment compared to dark-grown cells, consistent with rapid induction of ADV-1 levels in response to light stimuli (Figure 1, A and B). An overall reduction in the number of ADV-1 binding sites with longer light treatments (30 and 60 min) was observed, despite higher levels of ADV-1 protein accumulation in these samples. We speculate that this may be due to light-dependent posttranslational modifications of ADV-1 reducing its ability to bind to certain gene promoters. For the majority of light-regulated genes bound by ADV-1 (78%), ADV-1 binding changes in the light-treated samples, and light regulation was dependent on ADV-1 in about half of these genes (Figure 4). These data confirmed that ADV-1 signals light information to some of its downstream targets, supporting a hierarchical network of TFs controlling light responses (Chen *et al.* 2009). Alternatively, at some loci, ADV-1 binding occurred both in the dark and in the light, and the dark consensus-binding site differed slightly from the light bound ADV-1 consensus site (Figure S3), suggesting that ADV-1 functions in the dark to regulate distinct processes, including in relaying temporal information from the clock to downstream *ccgs*. Our data showing loss of rhythmicity for some ADV-1 direct targets confirm that ADV-1 is necessary to transduce temporal information to certain downstream *ccgs*.

Despite the major role of ADV-1 in light and circadian signaling, about half of the direct targets were predicted to be neither light- nor clock-controlled under our growth conditions. This may be due to mRNA stability precluding detection of changes in mRNA levels, or other modes of regulation, such as developmental control. In addition, ADV-1 binds to a site about 3 kb downstream of the *adv-1* transcriptional stop site, and our data support the idea that ADV-1 negatively regulates *adv-1* expression. Mathematical modeling demonstrated that negative autoregulation of the morning-specific CSP-1 repressor, a first tier transcription factor under direct control of the WCC, is necessary to buffer CSP-1-dependent oscillations of second tier evening-specific genes against fluctuations in the amplitude of the rhythms in WCC activity. This buffering capacity is thought to uncouple the amplitude of the second tier *ccgs* from the amplitude of the rhythms of the first tier genes (Sancar *et al.* 2011). Thus, similar to CSP-1, ADV-1 autorepression may be required for sustained robust rhythms of its downstream *ccg* targets. However, in both cases, this idea needs to be experimentally tested.

In *N. crassa*, cell-to-cell fusion plays an essential role in colony establishment, asexual development, and in the formation of protoperithecia (Fleissner *et al.* 2008). During colony establishment, CATs formed at the germ tube tips will fuse to establish an interconnected hyphal network. This branched hyphal network transports nutrients to aerial hyphae that, in turn, produce asexual conidiospores. Efficient transport of nutrients from the vegetative hyphae into developing protoperithecia is thought to be required for sexual development. Consistent with the role of cell-to-cell fusion in each of these processes, cell fusion mutants lack interconnected hyphal networks, produce short aerial hyphae and have reduced conidiospores, and they do not produce protoperithecia, rendering them female sterile (Lichius and Lord 2014; Read *et al.* 2012). Δ*adv-1* cells do not form CATs (Fu *et al.* 2011), and therefore, not surprisingly, the mutant has developmental defects that mimic other cell fusion mutants (Lichius and Lord 2014). Our genome-wide data are consistent with the phenotypes of the mutant, whereby the direct and/or indirect ADV-1 gene targets were found to function primarily in asexual and sexual development, hyphal growth, and metabolism. These data suggest coordinate regulation between genes involved in metabolism, growth, development, and cell fusion involving ADV-1. Changes in metabolism are likely required to accommodate the increased energy demands for development (Adomas *et al.* 2010). As ADV-1 is both light-induced and clock-controlled, and because light and the circadian clock influence the onset and timing of growth and development (Dunlap and Loros 2006; Corrochano 2007), our data point to a role for the clock, at least in part through ADV-1, in regulating cell fusion gene expression. Indeed, the cell fusion genes that are direct targets of ADV-1, with the exception of *pkr-1*, *ham-3*, *ham-5*, and *ham*-6, were found here (Figure 7) or previously identified (Table S8) as clock-controlled (Wu *et al.* 2014; Hurley *et al.* 2014; Lewis *et al.* 2002; Dong *et al.* 2008; Sancar *et al.* 2011, 2015b).

During cell fusion, chemotropic growth is associated with alternating oscillatory recruitment of the MAK-2 MAPK module (including ADV-1 targets MEK-1, MAK-1, and the scaffold protein HAM-5) and SO to opposing tips of the communicating cells prior to fusion (Jonkers *et al.* 2014, 2016; Dettmann *et al.* 2014, 2012; Fleissner *et al.* 2009b; Fleissner and Herzog 2016). This alternating pattern of MAK-2 complexes and SO at opposing tips occurs about every 4–5 min, and is thought to play an important role in signaling and response during fusion (Goryachev *et al.* 2012). HAM-3 (STRIATIN) and HAM-2 (STRIP) are required for fusion-related cell communication (Xiang *et al.* 2002; Fu *et al.* 2011; Simonin *et al.* 2010). In addition, they are necessary for assembly and function of the striatin-interacting protein phosphatase and kinase (STRIPAK) complex at the nuclear envelope (Dettmann *et al.* 2013). Components of the STRIPAK complex interact with components of the MAK-1 and MAK-2 MAPK pathways, suggesting that the STRIPAK complex functions to integrate the signals of the two MAPK pathways for cell communication required for fusion (Dettmann *et al.* 2013). HAM-7 was reported to function as a sensor for the MAK-1 MAPK pathway (Maddi *et al.* 2012), and HAM-6, HAM-7, and HAM-8 are required for MAK-1 kinase activation during conidial germination and CAT formation (Fu *et al.* 2014). HAM-9 is required for both MAK-1 and MAK-2 kinase activation in hyphae and is thought to play a role in communication between the two MAPK pathways during fusion (Fu *et al.* 2014). Based on sequence similarity to NoxD/Pro41 in *Podospora*, HAM-6 likely encodes p22phox of NADPH oxidases important in development, cell differentiation, cell proliferation, and programmed cell death (Lacaze *et al.* 2015). Furthermore, mutants of *N. crassa* lacking the NADPH oxidase NOX-1 are deficient in hyphal fusion (Fu *et al.* 2011; Fleissner *et al.* 2008), possibly due to connections between NOX complexes and MAPK pathways (Fleissner and Herzog 2016). Finally, both PRM-1 and LFD-1 function in membrane fusion (Fleissner *et al.* 2009a; Leeder *et al.* 2013; Palma-Guerrero *et al.* 2014). Taken together, our data suggest that ADV-1 signals temporal information from the clock to control rhythmic expression of cell fusion genes involved in all aspects of cell fusion, including chemoattraction, adhesion and cell wall remodeling, and membrane fusion.

Components of both the MAK-1 and MAK-2 MAPK pathways were found to be direct targets of ADV-1 (Table S3 and Figure S5); however, deletion of ADV-1 did not significantly alter the levels of mRNA for these MAPK components as determined by RNA-seq (Figure 6). CSP-1 potentially targets *mak*-2, but not the other MAPK genes, and no WCC or SUB-1 binding sites were identified for any of the MAPK genes (Smith *et al.* 2010; Sancar *et al.* 2011, 2015a) (Table S3). Taken together, these data further support the idea of combinatorial control whereby additional TFs may contribute to the regulation of the MAPK components. We previously reported that the circadian clock regulates daily rhythms in the phosphorylation and activity of MAK-1 and MAK-2 in *N. crassa* (de Paula *et al.* 2008; Lamb *et al.* 2011; Bennett *et al.* 2013; Vitalini *et al.* 2007); however, the contribution of ADV-1 to MAK-1 and MAK-2 activity rhythms has not been fully elucidated. The phenotypes of Δ*mek-1* and Δ*mak-1* deletion strains include defects in protoperithecia development, hyphal cell fusion, and asexual conidiation, similar to ΔADV-1 cells (Park *et al.* 2008, 2011; Fu *et al.* 2011). Although technically challenging, it is now of interest to determine if the clock controls the timing of cell fusion, as would be predicted based on rhythmic expression of the cell fusion genes and rhythmic MAK-1 and MAK-2 activity.

ADV-1 protein levels peak in the late day, several hours after the peak in WCC activity. We predicted that genes that are activated by ADV-1 would peak in mRNA levels close to the time of peak ADV-1 levels, and that genes that are repressed by ADV-1 would peak at the opposite phase, similar to what has been observed for genes controlled by the CSP-1 transcriptional repressor in *N. crassa* (Sancar *et al.* 2011, 2015b). However, our results are more complex. Specifically, *ham-9* is negatively controlled by ADV-1, and peaked in expression in the subjective early night (Figure 7). This is similar to the time when the mRNA levels peaked for the positively controlled *prm-1* and *ham-6* genes. Northern blot analyses to examine *ham-6* and *ham-9* mRNA levels over the course of the day confirmed the LUC reporter assays for these genes (Figure S9), ruling out discrepancies due to the use of the reporter. Furthermore, the residual rhythmicity of *ham-9* mRNA levels in ΔADV-1 cells cannot be explained by direct control of *ham-9* by the WCC, as no WCC was identified near the *ham-9* (Smith *et al.* 2010), nor simply explained by binding of SUB-1 and CSP-1 to the promoter of *ham-9* since both TFs also bind to the promoter of *ham-6* (Sancar *et al.* 2011, 2015a) (Table S3). Part of the explanation for these results may stem from the regulatory network downstream of ADV-1, including possible ADV-1 autoregulation, and other TFs that are direct targets of the WCC and/or direct targets of ADV-1. ChIP-seq revealed that many of the ADV-1 direct targets, including *adv-1* itself, are also targets of the other WCC-controlled TFs (Table S3) (Sancar *et al.* 2011, 2015a; R. Dekhang, *et al.*, unpublished results). These interactions form a nested set of feedforward and feedback loops that can have significant impacts on the dynamics of gene regulation (Kaplan *et al.* 2008; Alon 2007; Mangan and Alon 2003; Mangan *et al.* 2003). One function of the complex regulatory interactions that occur downstream of ADV-1 may be to generate distinct temporal dynamics of gene expression relative to the central clock, similar to what has been suggested for the clock- and light-regulated CPC-1 repressor in *N. crassa* (Sancar *et al.* 2011, 2015b). Taken together, our data support a model whereby, unlike the flat hierarchical network controlling light responses, the circadian regulatory network involves multiple clock-controlled TFs that coordinately bind to target gene promoters, and feed forward and back to control rhythmicity and phase.

In summary, using genome-wide approaches we demonstrate that ADV-1 plays a major role in transducing light and temporal information, as well as other unknown signals, to target genes involved in metabolism, cell growth, development, and cell fusion. These data provide an important resource for understanding the impact of light and the clock on regulating gene expression, and provides the platform for determining how interactions among TFs influence gene regulatory networks, including, for example, how cross-talk in the network controls the phase of rhythmic gene expression that is critical to temporal coordination.

## Supplementary Material

Supplemental material is available online at www.g3journal.org/lookup/suppl/doi:10.1534/g3.116.034298/-/DC1.
