# Supplementary figures and images for: The Neurospora Transcription Factor ADV-1 Transduces Light Signals and Temporal Information to Control Rhythmic Expression of Genes Involved in Cell Fusion
Source: G3 (Bethesda). 2016 Nov 15;7(1):129–42. doi: 10.1534/g3.116.034298 (PMC5217103; doi:10.1534/g3.116.034298)

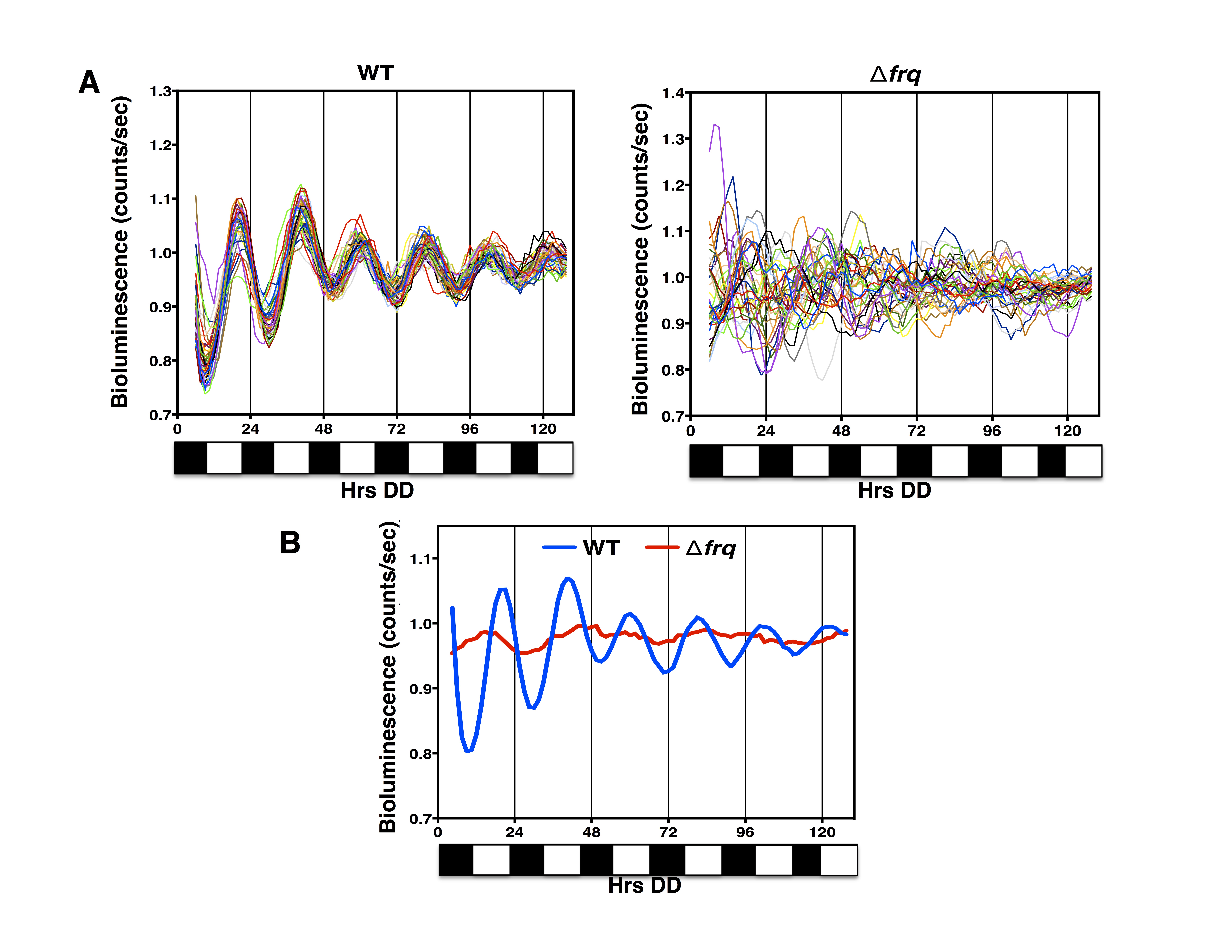

Supplement: Supplementary file 1 [file 129FigureS1.jpg]

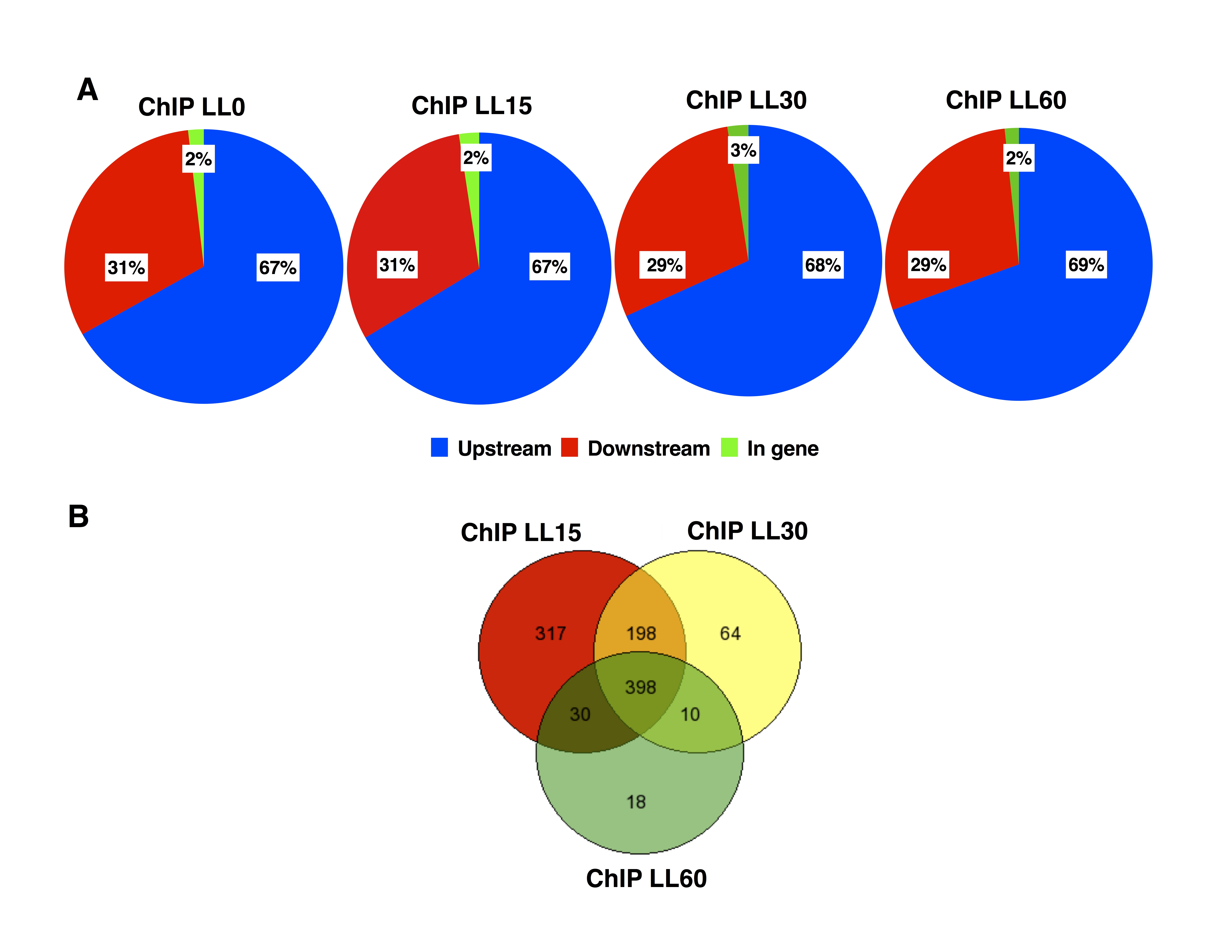

Supplement: Supplementary file 2 [file 129FigureS2.jpg]

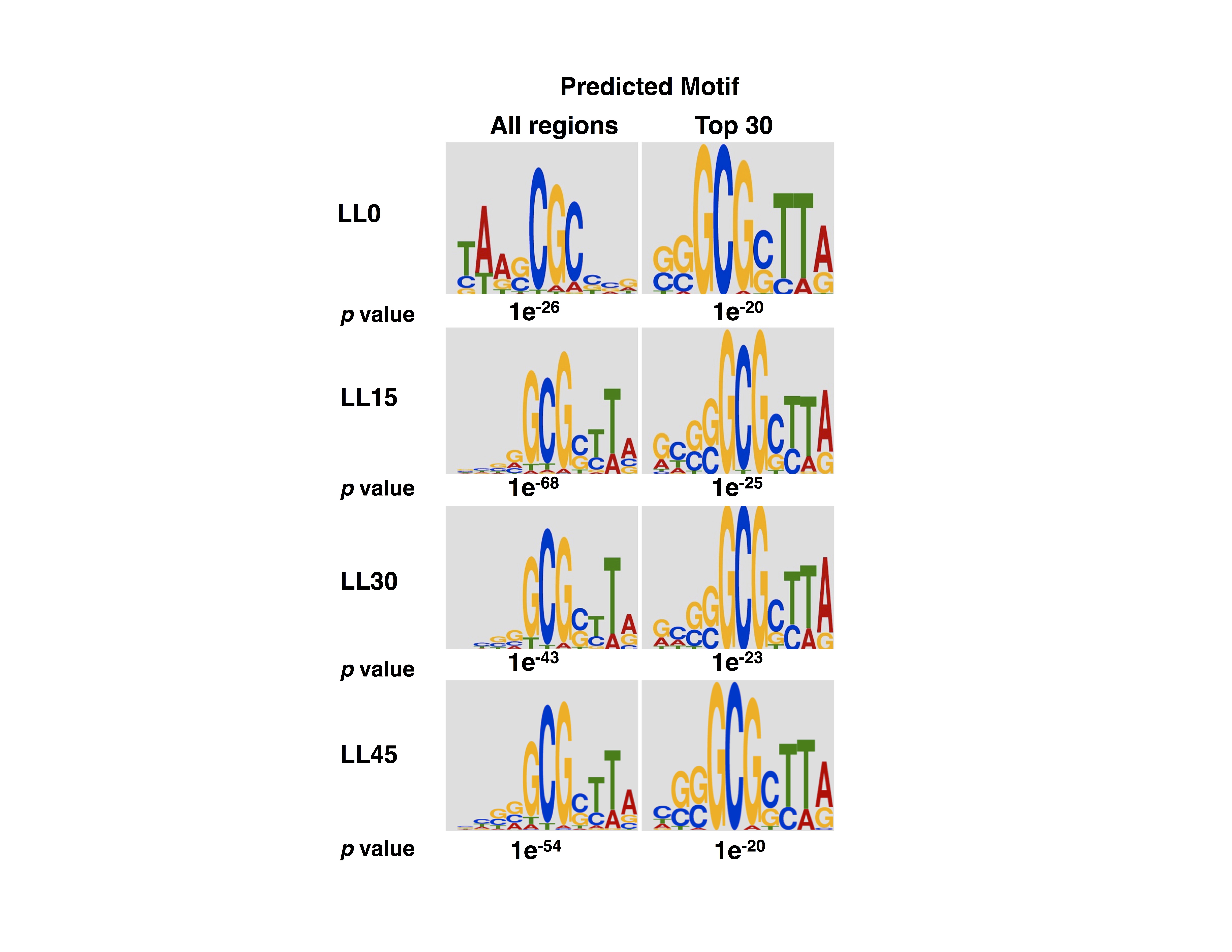

Supplement: Supplementary file 3 [file 129FigureS3.jpg]

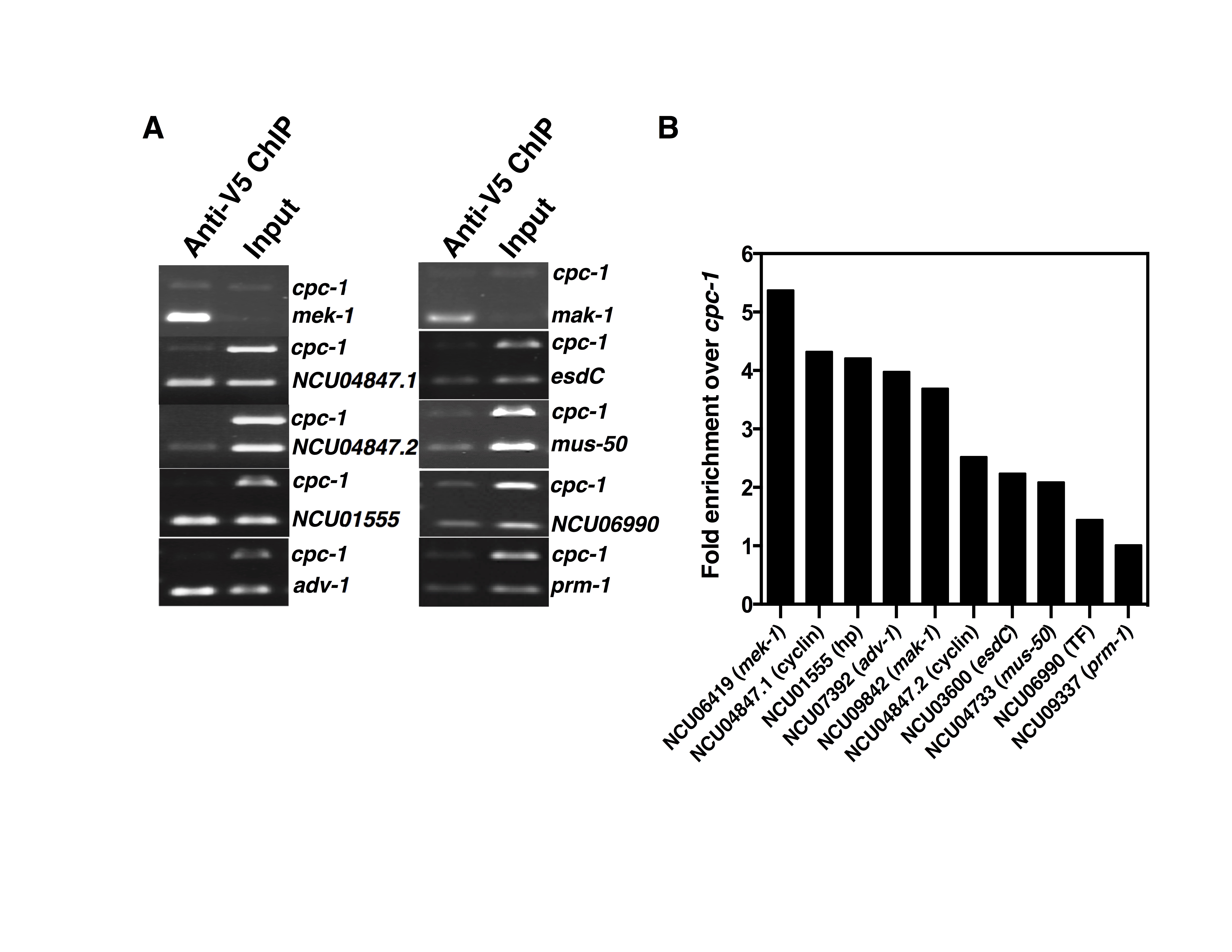

Supplement: Supplementary file 4 [file 129FigureS4.jpg]

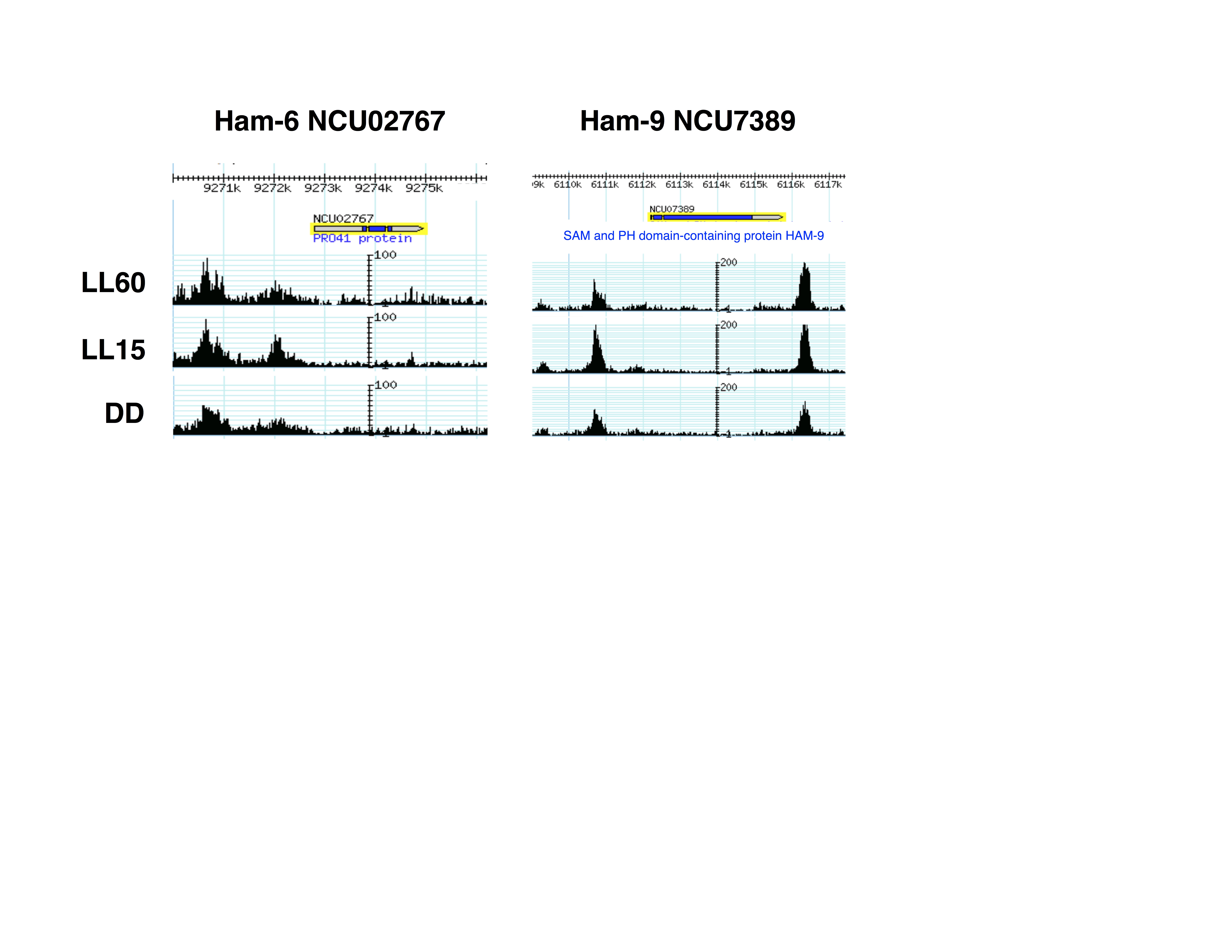

Supplement: Supplementary file 5 [file 129FigureS5.jpg]

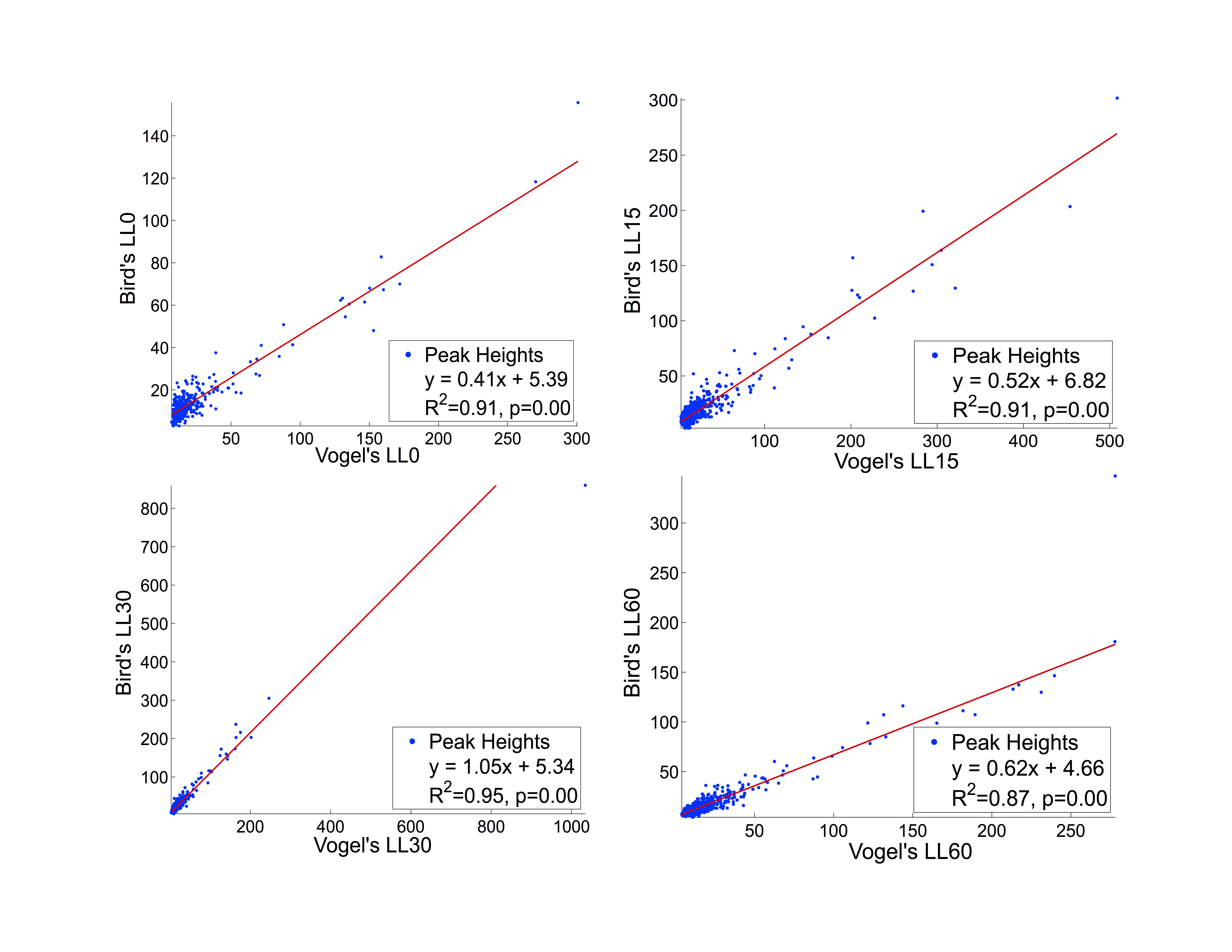

Supplement: Supplementary file 6 [file 129FigureS6.jpg]

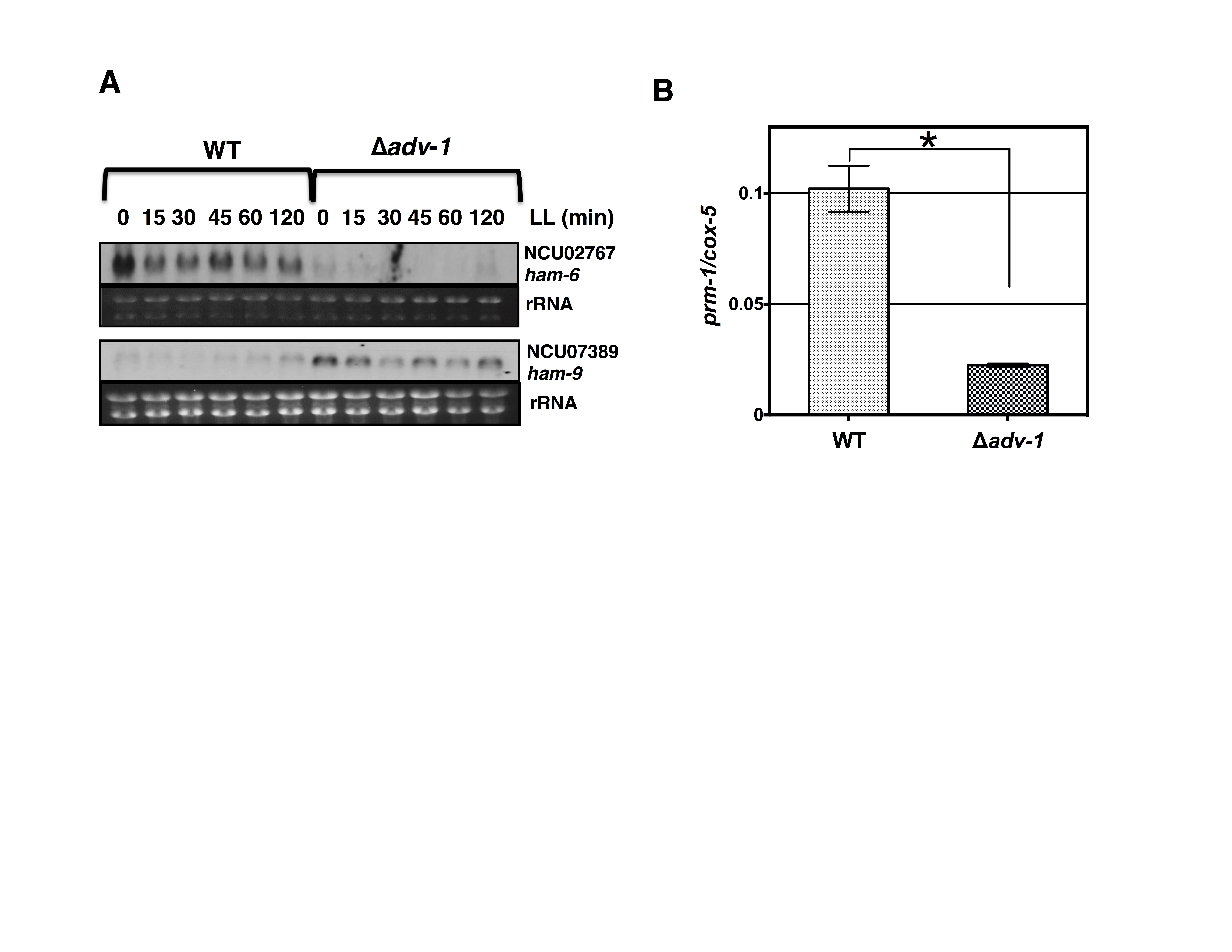

Supplement: Supplementary file 7 [file 129FigureS7.jpg]

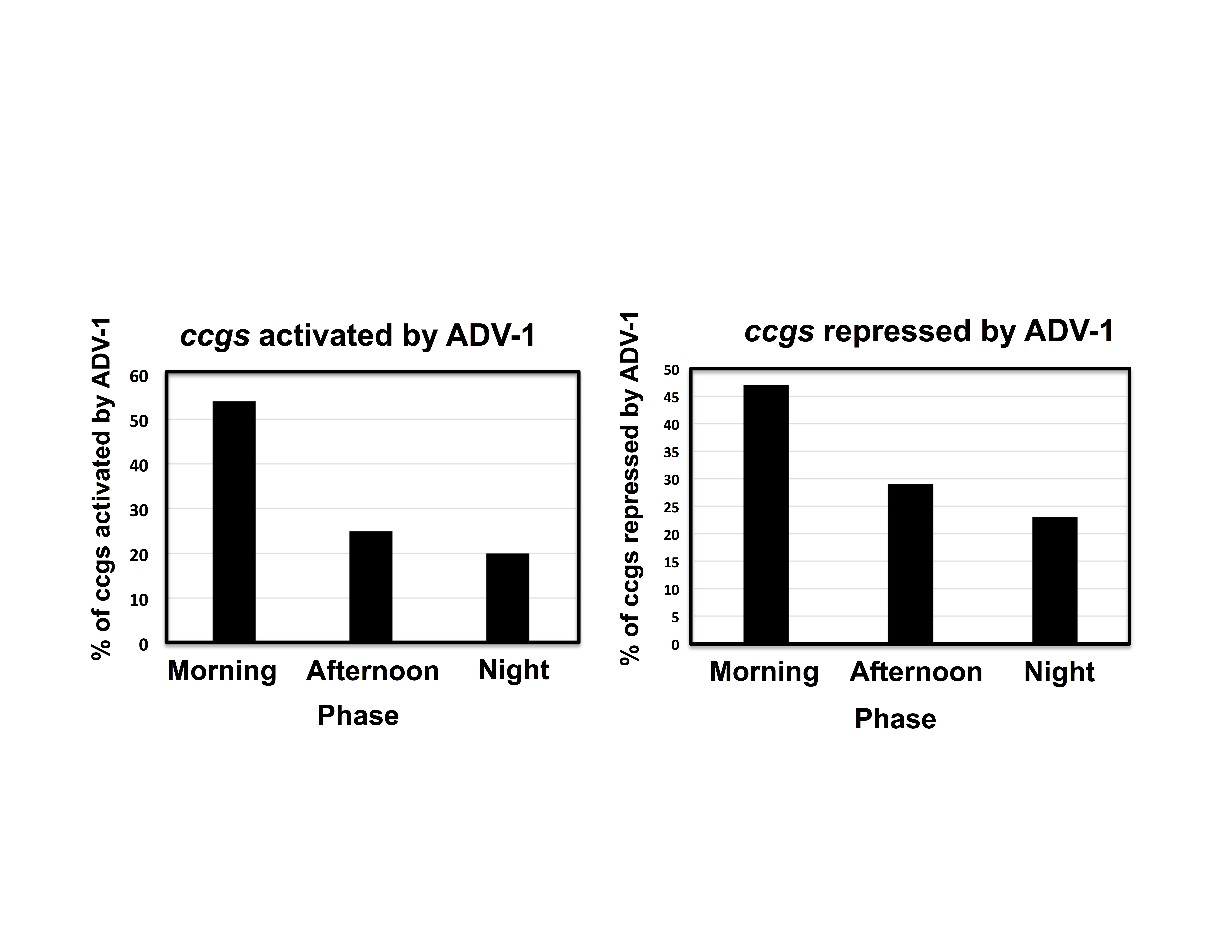

Supplement: Supplementary file 8 [file 129FigureS8.jpg]

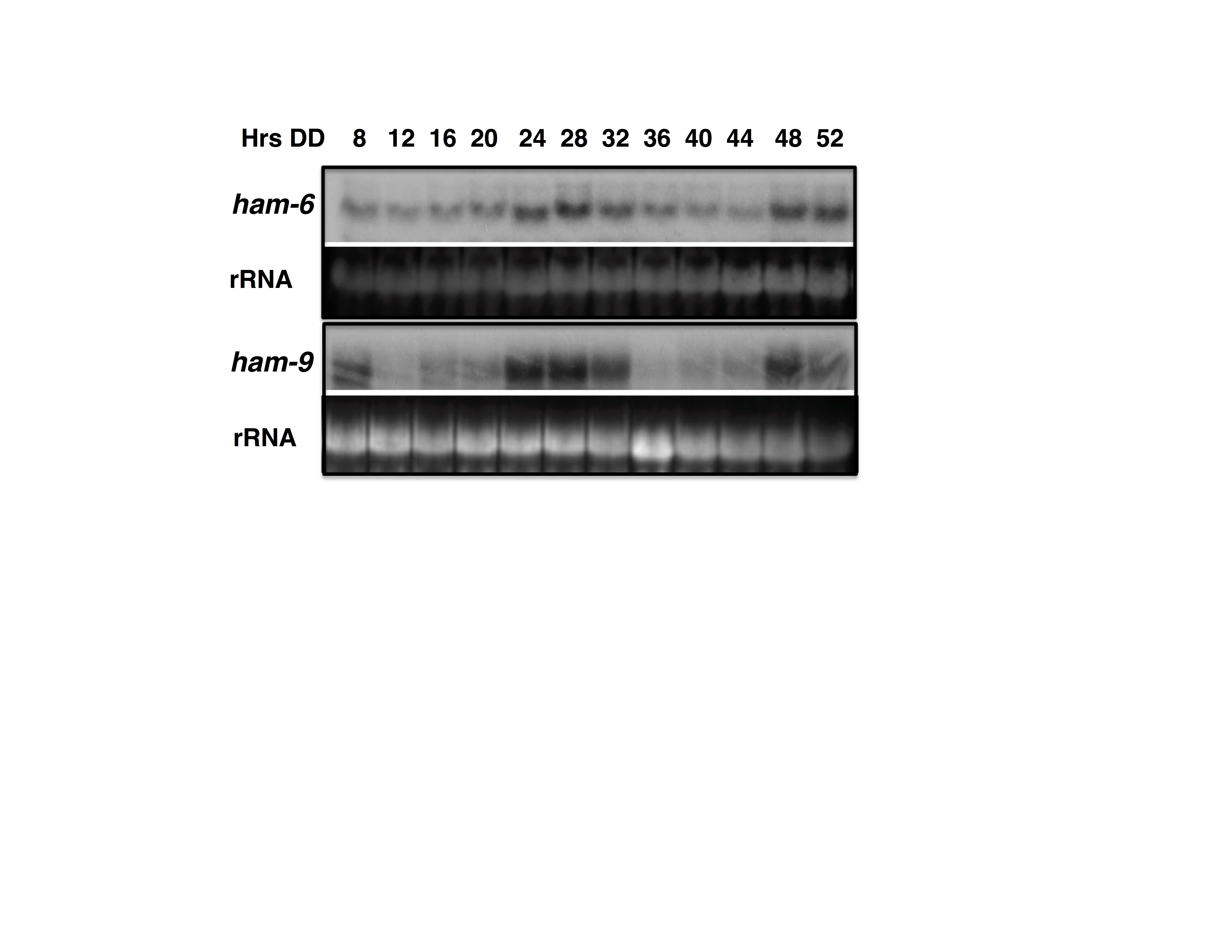

Supplement: Supplementary file 9 [file 129FigureS9.jpg]
