# Supplementary material for: The Neurospora Transcription Factor ADV-1 Transduces Light Signals and Temporal Information to Control Rhythmic Expression of Genes Involved in Cell Fusion
Source: G3 (Bethesda). 2016 Nov 15;7(1):129–42. doi: 10.1534/g3.116.034298 (PMC5217103; doi:10.1534/g3.116.034298)
Supplement: Supplementary file 10 [file 129FileS1.docx]

**Supplementary Figure Legends**

**Figure S1. ADV-1 protein levels are regulated by the clock. (**A) Individual bioluminescence recordings of ADV-1::LUC translational fusion in WT (n=42) (left), and Δ*frq* (n=34) (right) cells. (B) Average of all individual baseline detrended bioluminescence recordings of ADV-1::LUC in WT (blue) and ΔFRQ (red) cells. The time of recording in constant darkness (DD) is given on the x-axis (hrs). The bar graph below each plot represents subjective day (white) or subjective night (black) in DD.

**Figure S2. Overlap of ADV-1 binding sites and distribution of regions with ADV-1 binding (peaks) obtained by ChIP-seq. (A) Venn diagram showing the overlap of ADV-1 binding sites from cultures exposed to light for 15, 30, or 60 min. (B)** Pie charts show the distribution of ADV-1 peaks from cells that were harvested for ChIP after growth in the dark (LL0), and after the indicated times (min) in the light (LL). The categories are based on the position of the peak from the nearest transcriptional start site (TSS) of an annotated gene: upstream of the TSS (blue), downstream of the TSS (red) and within the transcript coding sequence (green). The number of peaks in each category divided by the total number of peaks for each time point are given.

**Figure S3.** **Predicted ADV-1 binding motifs.** ADV-1 consensus binding motifs were identified from the entire set of ADV-1-bound peak regions, and from the 30 most significant ADV-1-bound peaks under the indicated growth conditions for ChIP. The *p*-value for the motifs are indicated below.

**Figure S4.** **Validation of ADV-1 targets by independent ChIP-PCR.** (A) Ethidium bromide-stained gel of PCR products showing enrichment of ADV-1 bound signals at the promoters of the indicated genes compared to a negative control site *cpc-1* (anti-V5 ChIP). Input DNA served as a control for the ChIP (Input). For NCU04847, two different primer sets were used (NCU04847.1 and NCU04847.2) to validate ADV-1 peaks located at two different sites upstream of the gene (see Figure S5). (B) Plot of ADV-1 binding to gene promoters. The fold enrichment over input represents the amount of PCR product derived from amplification of ADV-1 target sites as compared to the negative control site *cpc-1* (n=1)*.* TF= predicted TF; HP= hypothetical protein.

**Figure S5.** Representative ADV-1::V5 ChIP-seq tracks for light- and ADV-1-regulated genes discussed in Figure 4, and ADV-1-regulated cell fusion genes discussed in Figure 6. The direction of transcription for each gene is shown by an arrowhead, and growth conditions for CHIP-seq are indicated on the left.

**Figure S6. Correlation of ADV-1-bound peak heights obtained from ChIP-seq performed in Vogel’s and Bird’s media.** The scatter plots show the coverage for peaks identified by ADV-1 ChIP-seq in Vogel’s medium (x-axis) against the coverage for peaks identified by ADV-1 ChIP-seq in Bird’s medium (y-axis) for each of the indicated growth conditions. Correlation coefficients (R^2^) are shown for each condition.

**Figure S7.** **Validation of RNA-seq data**. (A) RNA expression level analyses of the indicated cell fusion genes in WT and Δ*adv-1* strains. Northern blots of RNA from tissues harvested in the dark (0) and after the indicated light treatment (LL) in min. rRNA is shown as a loading control. (B) Total RNA isolated from WT and ∆*adv-1* cells given a 60 min light treatment was used for qRT-PCR to measure *prm-1* mRNA levels. The mRNA levels were normalized to *cox-5*, a gene not under the control of ADV-1 (± SEM, n=2). The asterisk represents statistically significant differences in *prm-1* mRNA levels in Δ*adv-1* cells as compared to WT cells (**p* <0.003, Student’s *t* test).

**Figure S8. No correlation exists between the phase of *ccg* expression and activation or repression by ADV-1.** Plots showing the percent of *ccgs* that are activated (left) or repressed (right) by ADV-1 on the y-axis and the phase in which the *ccgs* peak in mRNA accumulation (x-axis).

**Figure S9. mRNA levels of cell fusion genes are clock-controlled.** Northern blots of of mRNA isolated from WT cells grown in the dark (DD) and harvested every 4 h. The blots were probed with a *ham-6*- or *ham-9*-specific RNA probe. rRNA levels served as a loading control (n=1).
