# Supplementary material for: The Neurospora Transcription Factor ADV-1 Transduces Light Signals and Temporal Information to Control Rhythmic Expression of Genes Involved in Cell Fusion
Source: G3 (Bethesda). 2016 Nov 15;7(1):129–42. doi: 10.1534/g3.116.034298 (PMC5217103; doi:10.1534/g3.116.034298)
Supplement: Supplementary file 11 [file 129TableS1.docx]

Table S1. Primers used in this study. (.xlsx, 34 KB)

<http://www.g3journal.org/lookup/suppl/doi:10.1534/g3.116.034298/-/DC1/TableS1.xlsx>
